# Continuous Electrolyte Jet Scanning Enabled Near-Quantitative and High-Purity Silver Recovery from Silicon Solar Cells

**DOI:** 10.1021/jacsau.5c01469

**Published:** 2026-02-19

**Authors:** Wending Gu, David Payne, Shujuan Huang, Binesh Puthen Veettil

**Affiliations:** School of Engineering, 591223 Macquarie University, Sydney, 2109 New South Wales, Australia

**Keywords:** silver recovery, end-of-life silicon solar cells, electrolyte jet scanning, selective dissolution, mass-transport enhancement, closed-loop recycling

## Abstract

The accelerating retirement of end-of-life silicon solar cells (EoL-SSCs) is creating a rapidly expanding reservoir of secondary Ag, a critical yet finite resource, underscoring the urgent need for sustainable, high-efficiency recovery within a circular-economy framework. Conventional recycling routes are hindered by nonselective reactions, energy-intensive operations, and mass-transport bottlenecks. Here, we report a “top-down” electrolyte jet scanning (EJSC) strategy that enables selective, rapid Ag recovery while preserving the structural and functional integrity of the photovoltaic (PV) substrate. Tunable control of the interelectrode gap (IEG) and coordinated nozzle motion confines the reaction to a continuously refreshed microzone, allowing efficient and near-quantitative Ag extraction (97.1% in 4 min) from a 4 cm^2^ cell area under mild conditions (2 V, 12 wt % HNO_3_). Continuous interfacial renewal overcomes mass-transport limitations and produces kinetics that are significantly faster than those of static jet or bulk electrolysis systems. The dissolved Ag^+^ ions are directly reverse-electrodeposited with high efficiency (92.6% in 3 min) into Ag powders of 3*N*-level purity (99.88%), demonstrating potential as functional fillers for advanced electronics. EJSC also maintains high current efficiency throughout the operation (extraction, 89.6%; recovery, 79.6%) and sustains high Ag yield (>91%) over six repeated treatments, validating its strong industrial feasibility and scalability. Life-cycle and techno-economic assessments (LCA–TEA) further reveal that the process robustness of EJSC leads to markedly lower environmental impacts and significantly enhanced economic returns (>2500 USD/(kg of Ag)) relative to conventional processes. The confined-jet architecture inherently accommodates the thin, chemically sensitive passivation stacks used in emerging PV technologies. Integrating laboratory-level precision with industrial-scale throughput, EJSC delivers a closed-loop pathway for PV waste upcycling and establishes a versatile, sustainable platform for future urban mining of critical metals.

## Graphical abstract

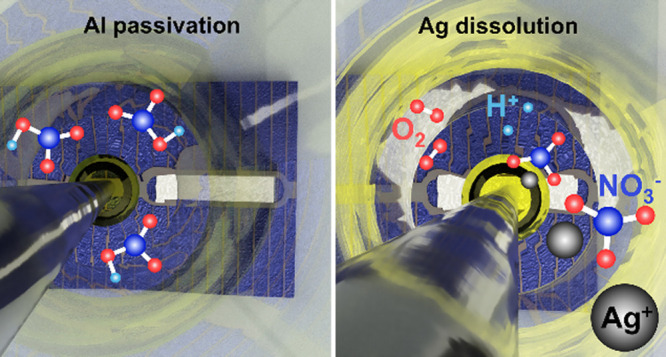

## 1 Introduction

The worldwide transition toward renewable energy has driven the large-scale deployment of photovoltaic (PV) technology, creating increasingly severe challenges in managing end-of-life (EoL) modules. By 2050, ∼80 million tons of crystalline Si solar cells (SSCs) are expected to be decommissioned,^1^ generating an unprecedented waste stream. These retired SSCs not only pose environmental risks but also constitute a valuable secondary resource. In particular, Ag in the front-contact metallization is the most critical component, with an estimated recyclable value of ∼$15 billion.^2^ Notably, the Ag content in SSC waste (∼700 g/ton) is comparable to that of primary Ag ores.^3^ However, mainstream hydrometallurgical recycling remains both economically and environmentally inefficient. These processes typically rely on concentrated HNO_3_ (≥5 M)^3–8^ leaching combined with complex displacement-precipitation steps to achieve targeted Ag recovery, which consume large amounts of reagents and risk coleaching of Al and other base metals. Motivated by sustainability concerns, recyclable deep eutectic solvents (DES) and nontoxic α-amino acids have been developed, enabling efficient Ag leaching (>96% yield) as soluble complexes, followed by precipitation as AgCl.^9,10^ In parallel, physical methods such as continuous-wave laser irradiation, pulsed laser debonding, and ultrasonic separation have been explored to directly liberate Ag microparticles from the Ag–Si interface,^11–13^ thereby reducing reagent consumption. Nevertheless, these approaches require elevated operating temperatures (≥80 °C) and extended processing times (up to 48 h), which limit their industrial deployment potential and economic viability. Consequently, there remains an urgent need for strategies that can achieve efficient and selective Ag recovery under mild conditions.

Against this backdrop, electrometallurgical recycling has rapidly emerged as a promising green strategy for Ag recovery,^14^ integrating efficient acid leaching with precise electrochemical extraction. Early demonstrations showed that Ag dissolved in HNO_3_ can be effectively recovered through Cu-assisted electrodeposition or redox displacement,^15,16^ which delivered rapid processing times (down to 80 min) and Ag recovery efficiencies exceeding 98%, thereby outperforming conventional hydrometallurgical routes in dissolution kinetics. These systems, however, also revealed several practical constraints, including the accumulation of concentrated Cu^2+^ in the electrolyte and the need for tightly regulated pulsed-potential cycles associated with sacrificial-metal dissolution and redeposition, which complicate process control and necessitate closed-loop effluent management. These limitations motivated the development of integrated dissolution–deposition strategies that avoid sacrificial metals altogether. For example, Deng and co-workers recovered ∼95% Ag by using the SSC itself as the anode in an Ag-plating electrolyte,^17^ while Yang, Lee, and co-workers utilized a metal-free methanesulfonic acid (MSA) electrolyte that enabled the formation of high-purity (>99.9%) dendritic Ag.^18,19^ Although these approaches simplified the reaction sequence and reduced reagent complexity, both remain constrained by diffusion-controlled Ag^+^ transport in a conventional electrolytic bath (E-bath), where the reaction interface is not actively controlled. Sustaining high reaction rates therefore requires either elevated cell voltages (≥24 V) or careful oxidant regulation (the MSA/H_2_O_2_ mixing ratio), underscoring the inherent limitation of bulk electrolysis and its inability to maintain a localized interfacial microenvironment necessary for selective and efficient Ag extraction from the heterogeneous architecture of EoL-SSCs.

Unlike conventional E-bath processes, which rely on passive electrolyte design and large-volume electrolyzers, miniaturized jet electrochemical machining (JECM) and jet electrodeposition (JECD) offer a directional route for Ag recovery with high spatial precision and enhanced mass-transfer efficiency.^20^ Both techniques employ ultrafine electrodes and microscale nozzles to deliver a high-speed electrolyte jet (EJ), generating highly localized reaction zones at the workpiece surface. JECM has been demonstrated for micromachining a wide range of metallic substrates, from Cu to hard-to-machine Ti alloys,^21,22^ whereas JECD has achieved ∼97.4% Cu^2+^ recovery from dilute wastewater streams with a current efficiency of 77.2%.^23^ Laser-assisted JECM has further shown the ability to induce highly localized dissolution in n-type Ge wafers,^24^ underscoring the broader potential of static electrolyte-jet (EJS) techniques for in situ extraction of conductive metals such as Ag from retired SSCs. However, EJS processes rely on fixed-nozzle configurations that cannot prevent reaction byproduct accumulation at the anode interface, resulting in nonuniform processing and parasitic corrosion. To alleviate interfacial deterioration, megasonic, ultrasonic, and Lorentz-force-assisted EJS variants have been explored to accelerate interfacial renewal and improve process uniformity.^25–27^ In reality, these auxiliary-field approaches may increase system complexity and energy demand while still failing to suppress byproduct buildup during long-term operation. Collectively, these persistent challenges highlight the intrinsic limitations of both EJS and E-bath systems, driving the innovation of the electrolyte jet scanning (EJSC) mode. By continuously refreshing the interfacial microenvironment and sustaining uniform ion transport under high-current conditions, EJSC not only enables precise fabrication of functional metals but also uniquely integrates localized metal removal with controlled redeposition for advanced surface modification.^28,29^ These considerations establish EJSC as a unified, streamlined platform that overcomes the mass-transport bottlenecks and interfacial instabilities constraining existing metal-recovery processes.

Here, we report an integrated EJSC strategy that enables selective, rapid, and near-quantitative Ag extraction (>97%) from spent SSCs under mild operating conditions (2 V, 12 wt % HNO_3_), surpassing EJS, E-bath, and conventional acid-leaching methods. The recycling system operates on a custom-designed 3D machining platform (Figure 1a), integrating a potentiostat for electrochemical analysis and a peristaltic pump for electrolyte recirculation, with the detailed setup provided in Supporting Information Figure S1. EoL-SSCs with typical front-contact architecture serve directly as the anode, with the extended Al busbar connected to the working electrode to minimize current interference from electrolyte splashing (Figure 1b). Localized electrolyte delivery establishes confined microreaction zones (Figure 1c), while the combination of a tunable interelectrode gap (IEG) and programmable nozzle scanning actively modulates reaction kinetics. Extraction selectivity is achieved through spatial confinement and precise exploitation of the oxidation-potential difference between Ag and Al (Figure 1d), allowing Ag to dissolve preferentially while Al busbars, fingers (Figure S2), and other non-Ag components remain intact (Figure 1e). Upon electrode-polarity reversal, liberated Ag^+^ ions are reduced into high-purity, highly conductive dendritic Ag powder (Figure 1f). Such a “top-down” recycling strategy enables an efficient and selective Ag recovery without downstream purification, thereby advancing sustainable PV-waste upcycling and closed-loop resource utilization within the broader paradigm of urban mining.

**Figure 1:**
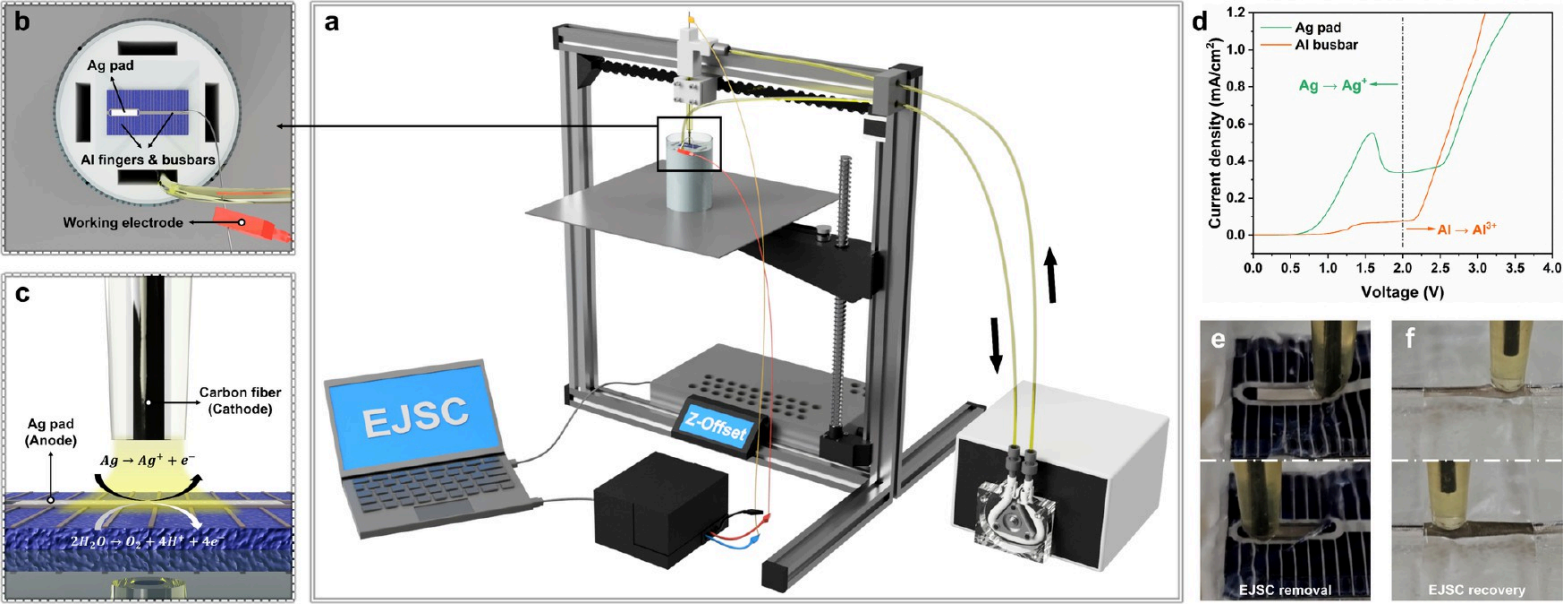
(a) Schematic of the EJSC/EJS recycling system. (b) Metal contact configuration in EoL-SSCs with working electrode assignments. (c) Microconfined Ag extraction principle. (d) LSV profiles (0–4 V, 10 mV/s), showing onset of Ag pad dissolution and Al busbar passivation at 2 V. (e, f) Photographs of Ag removal from SSCs and recovery onto coils during EJSC.

## 2 Results and Discussion

### 2.1 Limitations of EJS Relative to EJSC Recycling

In established electrolytic recycling systems,^17–19,30–32^ the applied voltage is typically regarded as the key parameter governing both dissolution rate and metal selectivity. EJS and EJSC, however, couple electrochemical driving forces with physical variables, thereby offering modes of control not attainable through applied potential alone. To illustrate this distinction, we first adopted the conventional strategy of increasing the voltage under static nozzle conditions (Figure 2a–d). At 2 V, optical imaging, scanning electron microscopy (SEM) and energy-dispersive X-ray spectroscopy (EDS) mapping revealed a heterogeneous response in which the Al busbar became passivated while dissolution was confined to the central region of the Ag pad (Figure 2b). Increasing the voltage to 4 and 6 V extended dissolution across the entire Ag pad, but this enhancement was accompanied by pronounced corrosion of the Al busbar (Figure 2c,d). These observations indicate that the applied voltage dictates both the spatial reach and intrinsic intensity of the electric field, which enhances dissolution but cannot simultaneously optimize Ag yield and selectivity. Motivated by this limitation and inspired by the dynamic jetting principles of inkjet 3D printing,^33^ we introduced an EJSC method. By translating the nozzle across the Ag pad at 6 mm/s, uniform Ag removal was achieved at 2 V, while the Al busbar remained essentially unoxidized in contact with the 12 wt % HNO_3_ jet (Figure 2e). Complementary results at higher voltages (Figure S3) show that EJSC, like EJS, exhibits reduced Ag selectivity under more aggressive conditions. By integrating electrochemical driving forces with nozzle motion, this dynamic strategy redefines EJ-based recycling as an actively tunable process rather than a passive response to applied voltage.

**Figure 2:**
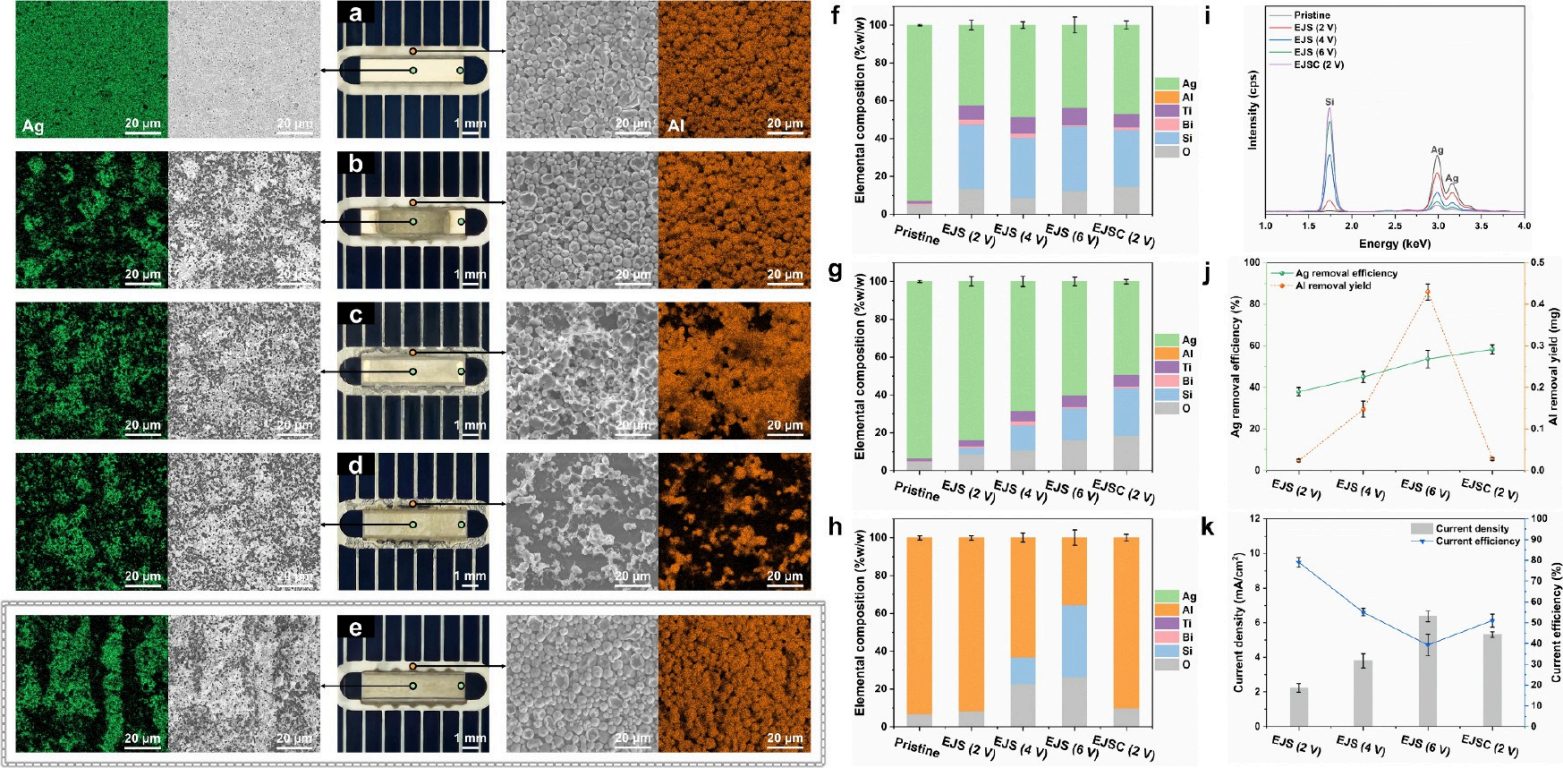
Comparative recycling performance of EJS and EJSC (IEG, 0.5 mm; scanning velocity, 6 mm/s; reaction time, 2 min). (a–e) Optical and SEM-EDS images of SSCs: (a) pristine; (b–d) EJS at 2, 4, and 6 V; (e) EJSC at 2 V. (f–h) EDS analyses of (f) Ag pad center, (g) Ag pad edge, and (h) Al busbar, showing residual elemental distributions. (i) XRF spectra of pristine and treated Ag pads. (j) ICP-MS quantification of Ag removal efficiency and Al dissolution yield. (k) Average current density and Ag-specific efficiency under EJS and EJSC. Error bars, where applicable, represent ±1 standard deviation (SD) from three independent trials (*n* = 3).

In addition to the central region shown in Figure 2a–e, complementary EDS maps collected at the pad edge (Figure S4) further corroborate spatial selectivity. From the quantitative EDS analysis (Figure 2f,g), the Ag content in the central region remains constant at 42–49% (w/w) under different voltages and nozzle motions, while the edge region decreases from 93 to 49% (w/w), demonstrating the spatial uniformity achieved by EJSC. Residual Al varies only with voltage and is unaffected by nozzle motion (Figure 2h), underscoring the protective effect of EJSC on Al components. In the X-ray fluorescence (XRF) spectra, the Ag Lα peak (∼2.98 keV) and Lβ peak (∼3.15 keV) attenuate, while the Si Kα peak (∼1.74 keV) intensifies (Figure 2i). This shift provides comprehensive spatial evidence for Ag dissolution across the entire contact. Inductively coupled plasma-mass spectrometry (ICP-MS) analysis (Figure 2j) further reveals a steady increase in Ag removal efficiency (eqs S3 and S4) from the static nozzle to the scanning nozzle at 2 V, confirming that the dissolved Ag^+^ exists in the recycled electrolyte as stable AgNO_3_. The maximum recoverable Ag content in the SSC is 2.5 mg (Table S1), determined by reference acid leaching in 70 wt % HNO_3_ for 48 h, which serves as the denominator in eq S4. The comprehensive Ag mass-balance data set (Table S2) in turn converts the spectroscopic observations into absolute mass values of dissolved and residual Ag. At 6 V under the static nozzle, Al dissolution rises sharply to 0.43 mg, indicating severe corrosion, whereas under scanning at 2 V it remains as low as 0.02 mg, demonstrating high Ag selectivity with minimal Al loss. Electrochemical performance analysis (Figure 2k) shows that, under the static nozzle, the current density of SSCs increases from 2.2 to 6.4 mA/cm^2^ as the voltage increases from 2 to 6 V, reflecting the strengthening of the electric field in accordance with Ohm’s law for cylindrical microelectrodes.^34^ In sharp contrast, EJSC achieves 5.3 mA/cm^2^ at only 2 V, comparable to that of EJS at 6 V. This behavior agrees with the finite element simulations of Oschätzchen and co-workers,^35^ which demonstrated that EJSC persistently renews the exposed surface, thereby suppressing local resistance accumulation during deeper etching. Additionally, the inverse relationship between current density and current efficiency (eqs S1 and S2) suggests that continuous scanning mode promotes electrolyte renewal and facilitates product removal, ultimately confirming the concurrent enhancement of both Ag extraction efficiency and energy utilization.

### 2.2 IEG-Controlled Current Density Defining Uniform Ag Extraction

Although the preliminary investigation revealed that EJSC achieved more than 50% Ag removal and current efficiencies within 2 min, we observed that excessive local current density degraded microscale etching quality, thereby limiting further improvement in overall extraction performance. To clarify this effect, we systematically investigated the role of the IEG in modulating current density and electric field distribution under constant voltage and nozzle-sample distance (Figure 3a). At an IEG of 0.5 mm, EDS mapping revealed pronounced longitudinal grooves on the Ag pad surface (Figure 3b), in agreement with Guo et al.,^36^ who showed that elevated current density accelerates material removal but fails to suppress microscale selective dissolution. These grooves primarily originated from heterogeneous dissolution driven by structural disparities in the Ag paste formed during high-temperature metallization. Zhou et al. reported that Ag particles generate silver oxide (Ag_2_O) during sintering, which disrupts bridging oxygen bonds in the glass frit, enhances fluidity, and produces a dense, low-resistance connection at the Si emitter interface.^37^ Such conductive domains serve as preferential pathways during extraction, and the narrow IEG led to elevated local current density that promoted selective dissolution at the Ag–Si interface. When the IEG was increased to 2.5 mm and 4.5 mm (Figure 3c,d), the groove structures gradually disappeared and were replaced by dispersed small aggregates. At 6.5 mm (Figure 3e), the aggregated regions expanded significantly. Consistently, EDS mapping at the Ag pad edges (Figure S5) revealed morphological evolution comparable to that observed at the pad center. These results indicate that an appropriate IEG of 4.5 mm fosters a synergistic coupling between electric field distribution and convection-enhanced mass transfer, which promotes uniform Ag extraction and alleviates diffusion-limited kinetics. At the optimum IEG, however, the kinetically activated dissolution simultaneously accelerates Al oxidation (Figure 3d).

**Figure 3:**
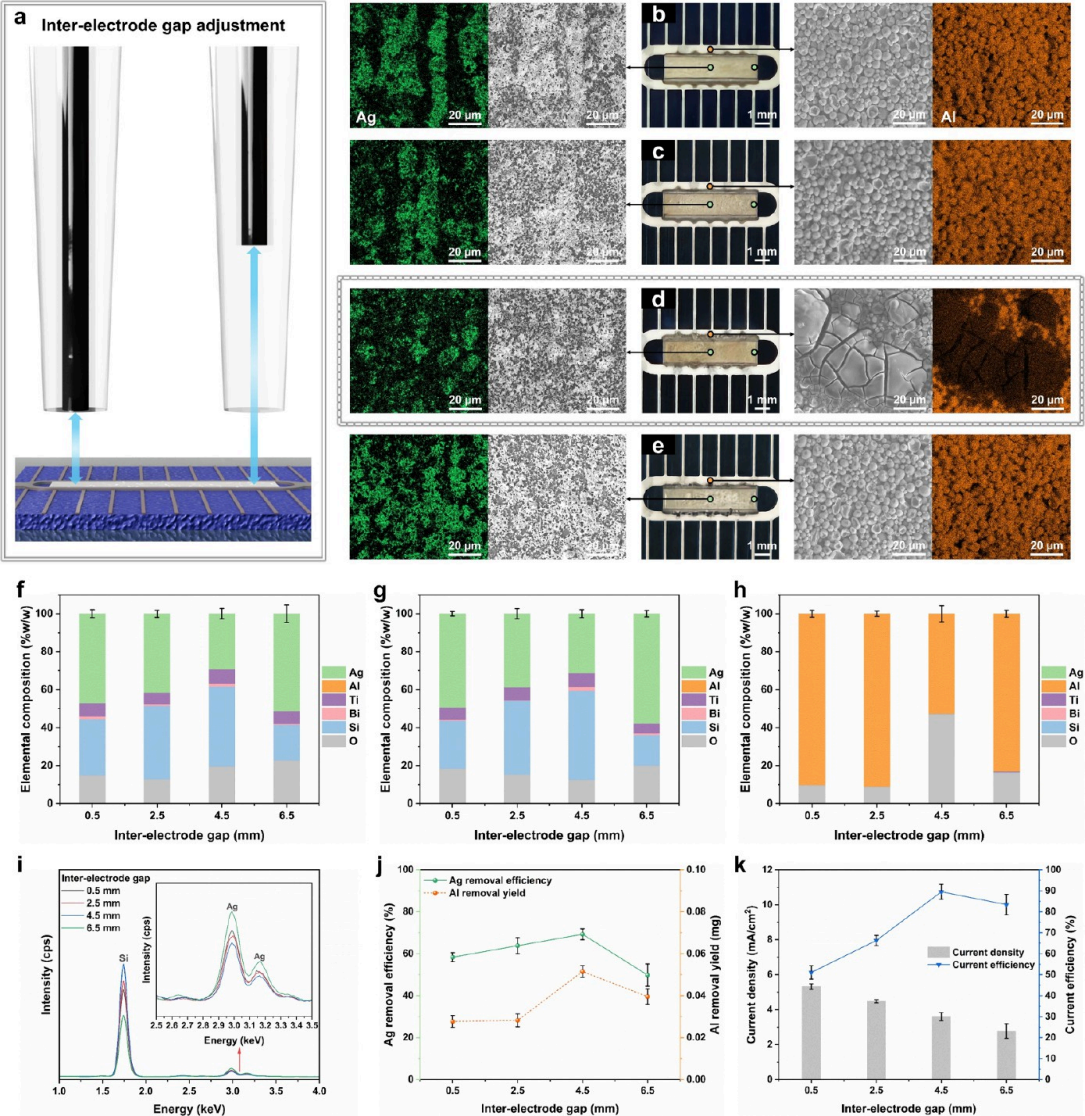
Effect of IEG on Ag removal uniformity and efficiency during EJSC (applied voltage, 2 V; scanning velocity, 6 mm/s; reaction time, 2 min). (a) Schematic of IEG variation. (b–e) Optical and SEM-EDS images of SSCs at IEGs of 0.5, 2.5, 4.5, and 6.5 mm. (f–h) EDS analyses of (f) Ag pad center, (g) Ag pad edge, and (h) Al busbar. (i) XRF spectra of Ag pads after EJSC. (j) ICP-MS quantification of Ag removal efficiency and Al dissolution yield. (k) Average current density and Ag-specific efficiency as a function of IEG. Error bars, where applicable, represent ±1 SD; *n* = 3.

Elemental analysis of the Ag pads (Figure 3f,g) shows that the residual Ag content follows a consistent trend across both central and edge regions under varying IEG conditions, confirming that the EJSC enables homogeneous Ag extraction on the macroscopic scale. A pronounced decrease in residual Ag content is observed as the IEG increases from 0.5 to 4.5 mm, accompanied by slight enrichment in Ti and Bi. These elements originate from the TiO_2_ antireflection coating and partial incorporation of Bi_2_O_3_ glass frit into the Si emitter, respectively, indicating exposure of these components without direct involvement in the reaction. At 4.5 mm IEG, the Al busbar exhibits only a distinct increase in O content (Figure 3h), without the widespread presence of Si signal observed under EJS at 6 V across the entire IEG range (Figure S6). In both recycling modes, a marked attenuation of the Al signal is detected, predominantly localized at the central junction with the Ag pad. These findings highlight that the optimized electric field distribution confines the microreaction zone in a highly localized manner, while refinement in fluid dynamics remains necessary to improve the overall Ag selectivity. When the IEG is further increased to 6.5 mm, Al oxidation is suppressed, most likely because the current density has fallen below its critical threshold. XRF spectra (Figure 3i) provide a global assessment of Ag removal across the pads. Except for a pronounced Ag peak at 6.5 mm IEG, spectra at other values show only minor deviations. Semiquantitative ICP-MS analysis (Figure 3j) further reveals that Ag removal efficiency increases steadily up to 4.5 mm IEG, but then drops to a level below that at 0.5 mm. This trend is consistent with the phenomenon reported by Dong and co-workers,^38^ where material removal rate decreases abruptly once the current density falls below a critical value. The same reversal is reflected in the electrochemical performance analysis (Figure 3k). Current efficiency is inversely related to current density up to 4.5 mm, but then declines in parallel with current density, from 89.5% to 83.4%, as the IEG increases to 6.5 mm. Collectively, these results demonstrate that excessive IEG causes a loss of electric field effectiveness, shifting Ag removal from active dissolution to passive oxidation and inducing oxygen evolution that reduces current efficiency.

### 2.3 Scanning Velocity-Driven Dynamics Governing Efficient Ag Extraction

In the EJS process, the reaction state resembles a still image, whereas in the EJSC process it is more akin to a dynamic sequence. Here, the nozzle velocity functions as a playback rate that must be fine-tuned to ensure smooth Ag extraction. At the optimal IEG of 4.5 mm, we examined how moderate increases in scanning velocity influence Ag dissolution (Figure 4a). EDS mapping (Figure 4b,c) reveals that increasing the velocity from 6 to 7 mm/s markedly reduced Ag signal intensity. Optical and SEM images confirmed extensive detachment of glass frit, exposing the silicon nitride (SiN_*x*_) passivation layer with visible cracks. At higher velocities (8–9 mm/s), the Ag signal intensity increased again, accompanied by the accumulation of larger and thicker frit (Figure 4d,e). Edge morphologies at different scanning velocities (Figure S7) corroborate the central-region trends. Notably, local frit accumulation was also observed at the pad edges when the scanning velocity reached 9 mm/s (Figure S7d). This nonmonotonic behavior mirrors prior JECM studies on reactive metals (e.g., stainless steel),^39,40^ where an optimal scanning velocity balances etching quality with suppression of stray corrosion. At low scanning velocities, prolonged electrolyte residence promotes stray currents, while excessively high scanning velocities shorten jet-substrate contact and weaken dissolution. For inert metals (e.g., Ti alloys),^41,42^ relatively low scanning velocities are generally required to disrupt the passive film and limit stray corrosion. In the present study, the Ag pad exhibited behavior consistent with the reactive-metal case, with Ag removal peaking at 7 mm/s and diminishing at higher velocities. By contrast, Al dissolution resembled the inert-metal scenario, in which lower scanning velocities facilitated stray corrosion. These findings delineate a narrow yet critical operational window, highlighting the importance of precise scanning velocity control for reliable Ag extraction.

**Figure 4:**
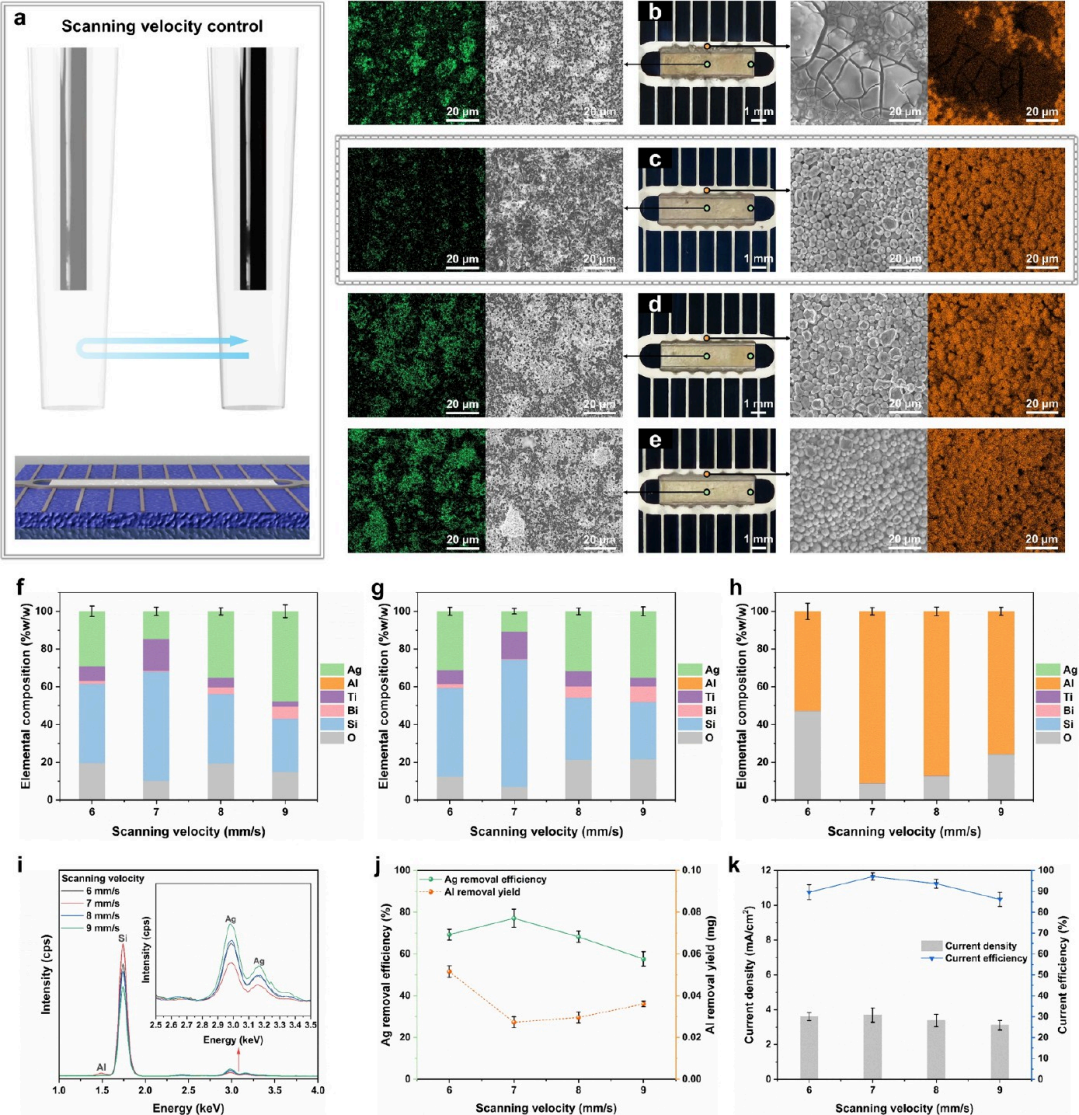
Effect of scanning velocity on Ag extraction during EJSC (applied voltage, 2 V; IEG, 4.5 mm; reaction time, 2 min). (a) Schematic of scanning velocity adjustment. (b–e) Optical and SEM-EDS images of SSCs at 6, 7, 8, and 9 mm/s. (f–h) EDS analyses of (f) Ag pad center, (g) Ag pad edge, and (h) Al busbar. (i) XRF spectra of Ag pads after EJSC. (j) ICP-MS quantification of Ag removal efficiency and Al dissolution yield. (k) Average current density and Ag-specific efficiency under different velocities. Error bars, where applicable, represent ±1 SD; *n* = 3.

Elemental profiles (Figure 4f,g) reveal that increasing the scanning velocity to 7 mm/s markedly reduces residual Ag across both the center and edge of the pad. At this velocity, the most prominent feature is a sharp rise in the Ti signal, originating from exposure of the TiO_2_ antireflection layer through fissures in the SiN_*x*_ passivation layer. At 9 mm/s, however, the center-edge disparity in Ag content expands to 13%, accompanied by a weaker Ti signal and Bi enrichment within the residual glass frit. These observations indicate that excessive scanning velocity compromises both the completeness and uniformity of Ag dissolution. A comparable trend was reported by Wang et al. in JECM of Ni-based alloys,^43^ where a moderate increase in velocity improved surface smoothness, though this effect was largely confined to the initial pass. Accordingly, even minor variations in scanning velocity during EJSC can disproportionately amplify changes in dissolution depth and frequency per unit time, yielding distinct morphologies and elemental distributions across the Ag pad. Compositional analysis of the Al busbar (Figure 4h) shows that residual Al content initially rises from 53 to 91% before declining, likely due to the broadened HNO_3_ jet footprint at higher velocities, which intensifies stray corrosion. This interpretation is consistent with Gao et al.,^39^ who found a direct proportionality between scanning velocity and machining width in JECM of stainless steels. XRF spectra (Figure 4i) further demonstrate that the residual Ag signal at 7 mm/s is only half that at 9 mm/s. ICP-MS quantification (Figure 4j) confirms an inverse correlation between Ag and Al dissolution, with Ag removal efficiency reaching 77.1% at 7 mm/s. The complementary mass-balance results (Table S2) additionally validate the reliability of the overall analysis across various EJ recycling conditions, with mass closure consistently within 100 ± 5%.

Consistent with these trends, electrochemical performance analysis (Figure 4k) further shows that at this velocity the current efficiency exceeds 95%, while the effect of scanning velocity on overall energy utilization is negligible compared with IEG. However, beyond 7 mm/s, both current density and current efficiency decline steadily, suggesting that stray current corrosion increasingly competes with the effective current available for Ag extraction. In addition to the kinetic control achieved through IEG tuning and scanning-velocity optimization, local mass transfer within the confined reaction zone is also shaped by key jetting parameters such as nozzle diameter and electrolyte flow rate. In our recent study,^44^ flow behavior in the 200–320 mL/min range was systematically examined using a fixed 2 mm nozzle that fully covered the 1.4 mm width of the Ag pad. A flow rate of 240 mL/min was identified as producing a stable transitional flow regime in which the thinnest diffusion boundary layer at the Ag–HNO_3_ interface was formed. Under this condition, the most uniform Ag dissolution and the lowest Al codissolution were achieved, while cavitation-induced field instability and current decay at higher flow rates were avoided. As a result, the combination of a 2 mm nozzle and a flow rate of 240 mL/min was established as the optimal mass-transfer environment. Under dynamic scanning, the coupling of the optimal jetting condition with the tuned kinetic parameters establishes a continuously renewed reaction interface and a stabilized local electric-field environment. These combined effects manifest as the high selectivity, rapid extraction kinetics, and excellent energy utilization that define the EJSC process.

### 2.4 Mechanistic Pathway Enabling Complete Ag Extraction and Reusable SSCs

To elucidate the fundamental mechanism underlying the superior Ag removal efficiency of the EJSC process compared with EJS and conventional bulk electrolysis in the E-bath, we systematically compared the temporal evolution of dissolution current under an identical IEG of 4.5 mm during a prolonged recovery period of 5 min. The EJSC current exhibited persistent and intense fluctuations over time (Figure 5a), whereas EJS displayed a more linear and steadier trend (Figure 5b). In contrast, the E-bath response (Figure 5c) resembled the overall current–time profile of EJS, although its maximum current remained far below the peak levels of both EJSC and EJS, reaffirming the intrinsic mass transport limitations of bulk electrolysis. Magnified views further revealed that EJSC initiated Ag dissolution within only 0.6 s, while EJS and E-bath required activation delays of 17.1 and 42 s, respectively, highlighting the pronounced kinetic lag in oxidative dissolution for the latter two regimes.

**Figure 5:**
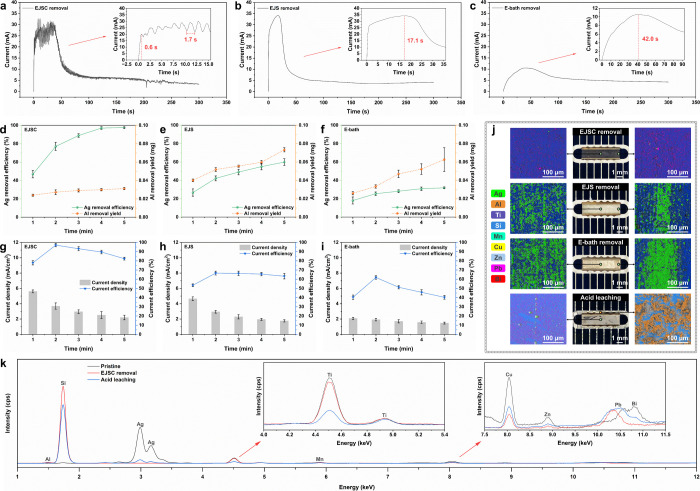
Electrochemical recycling behaviors of EJSC, EJS, and E-bath, with EJSC uniquely enabling practical SSC reuse through an efficient Ag extraction pathway (applied voltage, 2 V; IEG, 4.5 mm; scanning velocity, 7 mm/s; reaction time, 1–5 min). (a–c) Current–time responses during different processes. (d–f) ICP-MS quantification of Ag removal efficiency and Al dissolution yield as a function of time. (g–i) Time-resolved profiles of average current density and Ag-specific efficiency. (j) Optical and SEM-EDS images of SSCs after 4 min of electrochemical treatment by different strategies, compared with 1 h acid leaching in 5 M HNO_3_ (25 °C, 200 rpm). (k) XRF spectra of pristine, EJSC-treated, and acid-leached Ag pads. Error bars, where applicable, represent ±1 SD; *n* = 3.

The EJSC current oscillation period of ∼1.7 s precisely matched the nozzle reciprocation cycle of 6 mm at a scanning velocity of 7 mm/s, providing compelling evidence that Ag removal proceeds in a layer-by-layer fashion. When the reaction time was extended to 200 s, the EJSC current dropped markedly and lost its periodic order, suggesting near-quantitative removal of the Ag layer and the onset of stray current corrosion. In comparison, the currents of EJS and E-bath stabilized after an initial decline, which may arise from two factors. First, structural defects (e.g., deep grooves) formed during Ag extraction hinder the continuation of the reaction. Second, progressive dissolution of the Ag layer increases the distance between the newly exposed underlayer and cathode/nozzle, thereby elevating the dissolution resistance. Similar observations were reported by Bisterov et al. in JECM of Ni,^45^ where the resistance of the machining zone increased with IEG/nozzle-sample distance.

Quantitative ICP-MS analysis (Figure 5d) confirmed the prediction implied by the current–time response of the EJSC process, demonstrating that 97.1% of Ag was successfully extracted within ∼4 min, with the remaining 2.9% attributed to high-resistance regions formed during Ag paste annealing.^46^ With extended recycling, the Ag removal efficiency in EJSC increased at a progressively diminishing rate, whereas the dissolved Al content rose slightly from 0.024 mg to 0.031 mg. Extending the reaction time to 5 min therefore lifted the Ag removal efficiency marginally to 97.6%, although this was accompanied by the risk of stray corrosion of other components. In contrast, the dissolution of Ag and Al in EJS followed highly similar upward trends (Figure 5e), with Ag extraction limited to 59.9% after 5 min while Al dissolution nearly doubled. This lack of selectivity for Ag became even more pronounced in the E-bath process (Figure 5f), where Al dissolution increased markedly from 0.026 to 0.063 mg and Ag extraction efficiency dropped to less than half that of EJS. Macroscale EDS mapping (Figure 5j) further revealed the spatial differences in extraction performance among the three schemes. After EJSC processing, negligible Ag residue was detected in either the central or edge regions of the Ag pad, whereas EJS and E-bath treated samples exhibited extensive residual Ag accompanied by morphological defects that likely interrupted or terminated the dissolution reaction. Electrochemical performance analysis confirmed that EJSC delivered the highest overall current efficiency, sustaining 89.6% at 4 min, in contrast to the markedly lower values observed for EJS and the E-bath (Figure 5g–i). Notably, all three processes peaked at ∼2 min, indicating that Ag dissolution was concentrated in the early stage. In terms of current density, both EJS and EJSC exhibited a pronounced decline, while the E-bath showed little variation, reflecting its higher mass-transport resistance. Comprehensive elemental quantification across EJSC, EJS, E-bath, and a conventional acid leaching benchmark (5 M HNO_3_)^4,7^ widely adopted in hydrometallurgical recycling is summarized in Table S3. This comparison highlights the superior selectivity of EJSC, delivering the highest Ag yield among all electrochemical strategies and even surpassing the benchmark (2.43 mg vs 2.17 mg), while simultaneously suppressing metallic impurities by nearly 10-fold (0.08 mg vs 0.77 mg). Together, these results establish EJSC as the most effective strategy, integrating layer-by-layer dissolution with sequential scanning to achieve efficient and complete Ag extraction before electrochemical limitations become rate-determining.

Moreover, to highlight the core advantage of the EJSC process in safeguarding structural integrity of SSCs, we conducted a systematic comparison with the benchmark process. After 4 min of EJSC treatment, the front-side Al electrodes, including fingers and busbars, remained intact (Figure 5j). In stark contrast, 1 h of acid leaching under magnetic stirring at 200 rpm led to substantial dissolution of the Al metallization, and the Ag pad region exhibited extensive irregular cracking arising from the combined effects of mechanical agitation and excessive chemical corrosion. EDS elemental mapping further underscored the divergent outcomes of the two approaches. Conventional acid leaching removed the SiN_*x*_ passivation layer and severely corroded the underlying TiO_2_ antireflection coating, thereby exposing the Si substrate (light-blue signal). By comparison, EJSC largely preserved the SiN_*x*_/TiO_2_ bilayer (purple-blue signal), thereby establishing a robust basis for the subsequent reuse of SSCs. Effective acid leaching also requires prior mechanical disintegration of SSCs,^3,8^ including crushing, milling, and sieving to obtain particles with high specific surface area. Regeneration of Si wafers further relies on high-temperature pyrolysis combined with repeated etching in highly toxic hydrofluoric acid.^47^ Although these conventional methods achieve considerable Ag leaching efficiency and recover Si feedstock, they inevitably sacrifice the opportunity to reuse the valuable device architecture and impose complex processing steps.

In addition, full-spectrum XRF analysis (Figure 5k) corroborates the chemical selectivity of EJSC. In the Ag pad region, EJSC treatment results in complete removal of Ag, whereas residual signals remain detectable in acid-leached samples. The magnified spectra on the left show that the Ti Kα (∼4.51 keV) and Kβ (∼4.93 keV) peaks of the EJSC-treated SSCs are nearly identical to those of the pristine device, confirming that the TiO_2_ antireflection layer is essentially preserved. The enlargement on the right further reveals that only elements with high reactivity in HNO_3_, such as Cu Kα (∼8.05 keV), Zn Kα (∼8.64 keV), and Bi Lα (∼10.84 keV), exhibit reduced intensities under EJSC conditions, underscoring the enhanced electrochemical activity of metals in a forced convection environment. Meanwhile, the Pb Lα peak (∼10.55 keV) undergoes a slight downshift in energy consistent with partial reduction of PbO,^48,49^ indicating that the Pb-containing additives used to lower the glass frit sintering temperature remain intact. Trace elements such as Mn, which facilitate charge transport and suppress surface recombination,^50^ also retain signal intensities comparable to those of the original SSCs. In sharp contrast, acid-leached SSCs display marked attenuation of the Ti peaks, providing direct evidence of pronounced corrosion of the TiO_2_ layer, while the overlapping Pb Lα and Bi Lα features broaden and shift to lower energy, reflecting chemical state transformations rather than dissolution in 5 M HNO_3_. Collectively, these spectral results demonstrate that conventional hydrometallurgy relies on nonselective reactions in concentrated acids, lacks effective control over functional layers and impurity elements, and therefore exhibits inherent limitations.

Importantly, complementary current–voltage (*I*–*V*) measurements and passivation-related parameters (Figure S8) subsequently highlighted that EJSC-treated SSCs retained intrinsic PV functionality. The device exhibited only a slight reduction in short-circuit current (*I*_sc_) due to Ag removal, while maintaining a stable open-circuit voltage (*V*_oc_), fill factor (FF), and shunt resistance (*R*_sh_), resulting in a comparable power conversion efficiency (PCE) of 3.5% to the pristine cell (4.2%). Notably, the *I*–*V* curve preserved its characteristic plateau, underscoring intact charge transport and passivation quality. Despite the nearly unchanged *V*_oc_, acid-leached samples suffered a drastic loss of *I*_sc_ and collapse of the *I*–*V* plateau, accompanied by reduced FF, elevated *R*_sh_, and a sharply degraded PCE of 0.4%, indicating pronounced passivation failure and functional loss. Overall, the EJSC process achieves targeted Ag extraction while safeguarding functional metals and device integrity, charting a sustainable and forward-looking pathway for the reuse of EoL-SSCs.

### 2.5 Electrochemical Kinetics Underlying Distinct Ag Extraction Techniques

The superiority of EJSC in macroscopic Ag extraction arises from its distinctive interfacial reaction kinetics. Cyclic voltammetry (CV) analysis reveals fundamental differences in the double-layer behavior across the three strategies (Figure 6a–c). Within the non-Faradaic potential region, all systems exhibited ideal capacitive responses as the scan rate increased from 10 to 50 mV/s, while the potential window and curve morphology varied markedly. EJSC produced fluctuations in the CV curves due to high-velocity EJ perturbation (Figure 6a), but it maintained the broadest non-Faradaic window of 0.7 V vs standard hydrogen electrode (SHE). This wide window indicates that the dynamically refreshed interfacial microenvironment effectively removes surface-adsorbed and near-surface species, such as Ag^+^ intermediates,^51^ nitrate-derived nitrogen oxides,^52^ and metallic impurities, thereby suppressing side reactions. In contrast, EJS was constrained by a locally stagnant microenvironment, where byproduct accumulation led to current density depression at more negative potentials and deterioration of curve symmetry, narrowing the CV window to 0.4 V (Figure 6b). The static E-bath system, limited by bulk-phase mass transport, exhibited a severely compressed window of only 0.1 V (Figure 6c), reflecting pronounced enrichment of parasitic species at the interface. Quantitative analysis of double-layer capacitance (Cdl) confirmed this trend (Figure 6d), with values decreasing from EJSC (386.6 μF/cm^2^) to EJS (356.0 μF/cm^2^) and E-bath (122.7 μF/cm^2^). Based on a representative specific capacitance of 40 μF/cm^2^,^53^ the corresponding electrochemically active surface areas (ECSA) were calculated, with EJSC reaching 38.7 cm^2^, indicating the greatest abundance of accessible active sites. These parameters capture only time-averaged activity, with the instantaneous ECSA of EJS (∼35.6 cm^2^) appearing close to that of EJSC. The true advantage of EJSC, however, emerges under dynamic extraction, where continuous interfacial renewal sustains an overall ECSA far beyond the range attainable in static recycling systems.

**Figure 6:**
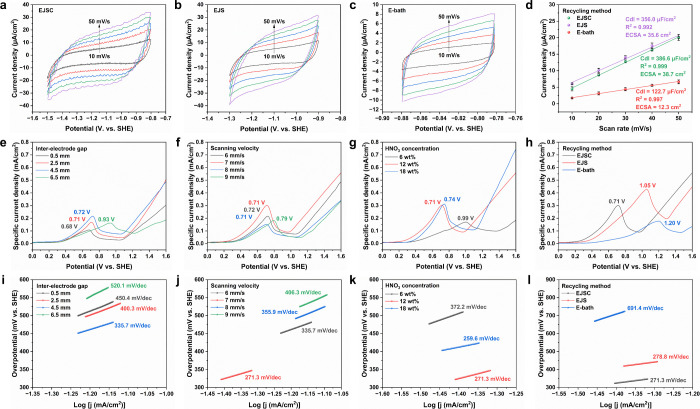
Interfacial electrochemical kinetics and mass transport during Ag extraction. (a–c) CV curves (10–50 mV/s vs SHE) for (a) EJSC, (b) EJS, and (c) E-bath. (d) Comparison of Cdl and ECSA. (e–g) LSV profiles of EJSC showing effects of (e) IEG, (f) scanning velocity, and (g) HNO_3_ concentration. (h) Comparative LSV curves of EJSC, EJS, and E-bath. (i–k) Tafel plots of EJSC under varied conditions: (i) IEG, (j) scanning velocity,and (k) HNO_3_ concentration. (l) Comparative Tafel plots of EJSC, EJS, and E-bath under optimized conditions (IEG, 4.5 mm; scanning velocity, 7 mm/s; HNO_3_ concentration, 12 wt %). Error bars, where applicable, represent ±1 SD; *n* = 3.

To establish the connection between interfacial kinetic features and Ag extraction efficiency, systematic linear sweep voltammetry (LSV) studies were further conducted to elucidate the role of mass transport regulation in governing macroscopic reaction rates. In IEG optimization (Figure 6e), increasing the spacing from 0.5 to 4.5 mm markedly enhanced the limiting current density (i.e., specific current density normalized to ECSA), indicating that expansion of the reaction zone effectively strengthened interfacial mass transport, and the peak potential shifted from 0.68 to 0.72 V, reflecting a transition from localized, nonuniform dissolution under high current density to a more homogeneous and controllable mass-transport-dominated process. When the IEG was further enlarged to 6.5 mm, the peak potential rose to 0.93 V and the limiting current decreased, demonstrating that excessive spacing weakened the electric field and restricted mass transport, thereby imposing severe kinetic limitations. Optimization of scanning velocity (Figure 6f) revealed that a velocity of 7 mm/s yielded the maximum limiting current density, highlighting an optimal balance between EJ impingement frequency and Ag dissolution rate. At a lower velocity of 6 mm/s, prolonged residence time promoted side reactions, whereas at a higher velocity of 8 mm/s, insufficient contact reduced dissolution efficiency. At 9 mm/s, the peak potential shifted positively to 0.79 V with uneven Ag removal and agglomerated glass frit (Figure 4e), confirming that excessive velocity drives the process into an inefficient regime.

Beyond spatial and kinematic operational parameters, electrolyte concentration emerged as another critical factor influencing interfacial kinetics and extraction efficiency. The LSV curves (Figure 6g) revealed that increasing the HNO_3_ concentration from 6 to 12 wt % significantly enhanced the limiting current density, with the peak potential shifted markedly from 0.99 to 0.71 V. This shift indicates that at lower acidity the reaction is constrained by insufficient proton supply and limited ionic strength, which impose substantial mass-transport resistance and necessitate a high driving potential, whereas 12 wt % HNO_3_ provides an optimal initial chemical driving force that enables efficient reaction onset at lower potentials. Although 18 wt % HNO_3_ generated the highest limiting current, the peak potential shifted slightly to 0.74 V, suggesting that the strongly corrosive environment may trigger undesirable side reactions that compromise selectivity. Direct comparison of the three strategies (Figure 6h) further underscored the superiority of EJSC, which achieved efficient Ag dissolution at the lowest driving potential of 0.71 V, while EJS (1.05 V) and the E-bath (1.2 V) required substantially higher potentials. This distinction reflects the fundamental difference in mass-transport efficiency, since EJSC relies on a dynamically refreshed interfacial microenvironment to sustain efficient reactions under mild conditions, whereas the static recycling systems remain constrained by mass transport bottlenecks and depend on stronger driving forces to overcome kinetic barriers.

To quantitatively decipher the kinetic origins of electrochemical behaviors, Tafel slope analysis was employed to delineate the microscopic kinetic boundaries governing Ag extraction performance and energy consumption. With the increase of IEG from 0.5 mm to 4.5 mm (Figure 6i), the Tafel slope dropped significantly from 450.4 to 335.7 mV/dec (overpotential window shifted cathodically from 500–540 to 450–480 mV). While the slope still exceeds the theoretical limit for a one-electron transfer (∼120 mV/dec),^54,55^ enlarging the IEG strengthens convective mass transfer and drives the reaction mechanism from a severely mass-transfer-constrained regime toward a mixed kinetic-mass-transfer region. Optimization of the scanning velocity (Figure 6j) further revealed that an optimal slope of 271.3 mV/dec was achieved at 7 mm/s (320–350 mV), suggesting optimal synergy between EJ-induced convection and interfacial reaction kinetics under this condition. When the scanning velocity was further increased to 8 and 9 mm/s, the Tafel slope rose to 355.9 and 406.3 mV/dec (overpotential windows 500–530 mV and 530–560 mV), indicating that excessively high scanning velocities lead to insufficient EJ-substrate contact time, thereby reducing the efficiency of reactant replenishment and product removal and shifting the reaction to a mass-transfer-dominated regime. Electrolyte concentration studies revealed more complex interfacial behavior (Figure 6k). Under proton-deficient conditions with 6 wt % HNO_3_, the reaction exhibited a slope of 372.2 mV/dec (480–510 mV), indicating that insufficient proton supply constituted the primary kinetic bottleneck. When the concentration was increased to 18 wt %, the high proton flux further reduced the slope to 259.6 mV/dec (400–420 mV), yet paradoxically shifted the overpotential window anodically, suggesting that the strongly corrosive environment may induce restructuring of the Ag-HNO_3_ interfacial double layer and intensify nonselective corrosion, thereby partially offsetting the kinetic benefits of enhanced mass transfer. A systematic comparison of different Ag extraction strategies further clarified the kinetic boundaries (Figure 6l). EJS exhibited a Tafel slope of 278.8 mV/dec (420–440 mV), revealing the intrinsic restriction of local microenvironmental stagnation. The E-bath displayed an extremely high slope of 691.4 mV/dec (670–720 mV), quantitatively reflecting the severe mass-transfer limitation inherent to conventional bulk electrolysis. Consequently, these kinetic parameters verify that the EJSC method under optimized conditions (4.5 mm IEG, 7 mm/s scanning velocity, and 12 wt % HNO_3_) facilitates a smoother kinetic pathway, thereby enabling near-quantitative Ag dissolution on an ultrafast time scale.

### 2.6 Closed-Loop Recovery of Commercial-Grade Ag via Reverse Electrodeposition

The high selectivity achieved in Ag extraction provides a robust foundation for closed-loop resource recovery, and the efficient electrodeposition of dissolved Ag^+^ into high-purity metallic Ag constitutes the critical step in transforming waste into wealth. Using the recovered electrolyte from the above-described 4 min EJSC Ag extraction step, we systematically evaluated the electrodeposition behaviors of EJSC, EJS, and the E-bath on Ag coil cathodes at 2 V via electrode polarity reversal. The sequence of Ag recovery efficiencies closely mirrored their extraction performance (Figure 7a), confirming that interfacial mass transport is the key determinant of the overall recovery process. At the early stage of deposition (1 min), EJS exhibited a markedly higher recovery efficiency (eq S5) of 45.5%, compared with 29.4% for EJSC. This behavior stems from the fixed nozzle in EJS, which establishes a stable localized reaction zone that promotes Ag^+^ supersaturation, thereby lowering the nucleation barrier and accelerating Ag nucleation. In contrast, although the high-velocity EJSC continuously generates new nucleation sites, its vigorous perturbation disperses local Ag^+^ ions and destabilizes the stability of the concentration boundary layer, ultimately inhibiting initial nucleation. As deposition progressed, the dynamic interfacial renewal and superior mass-transport characteristics of EJSC progressively dominated the process, achieving a recovery efficiency of 93.6% within 5 min, markedly higher than the 77.1% obtained with EJS. By comparison, the E-bath system, constrained by limited ionic diffusion, consistently exhibited low recovery efficiency, with a maximum of only 15.4%.

**Figure 7:**
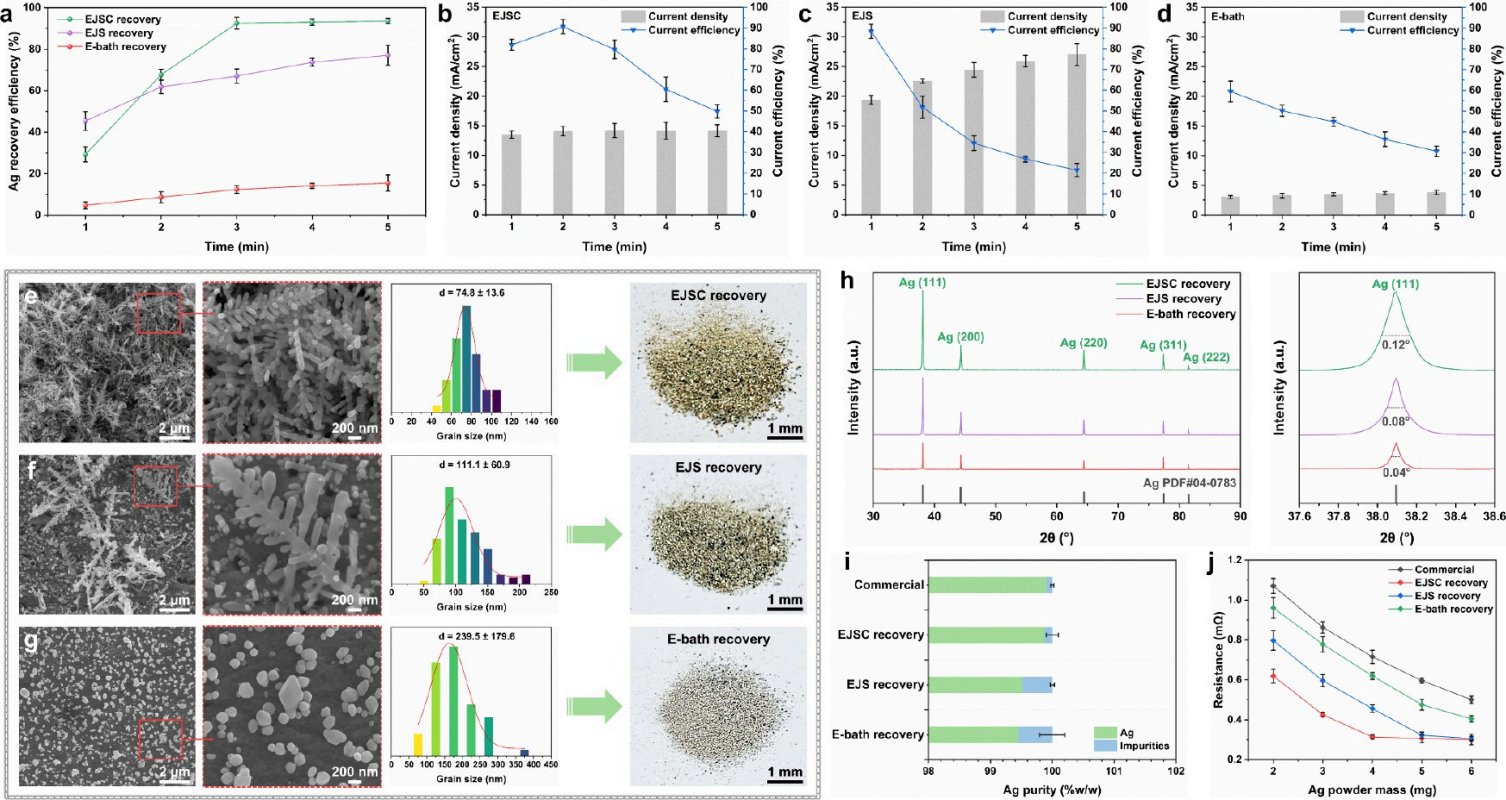
Closed-loop Ag recovery by reverse electrodeposition using EJSC, EJS, and E-bath (applied voltage, 2 V; IEG, 4.5 mm; scanning velocity, 7 mm/s). (a) Time-dependent Ag recovery efficiencies. (b–d) Evolution of average current density and efficiency. (e–g) SEM morphologies and particle size distributions of recovered Ag, with photographs of powders after three cycles. (h) XRD patterns of recovered Ag compared with standard Ag PDF card, with enlarged Ag(111) reflection. (i) Purity of commercial 3N–Ag and recovered Ag. (j) Bulk resistance as a function of mass loading for Ag powders recovered by the three methods. Error bars, where applicable, represent ±1 SD; *n* = 3.

Electrochemical performance further underscores the mechanistic distinctions among these strategies. EJS exhibited a remarkably high current efficiency of 88.4% within the first minute, accompanied by a continuous increase in current density over time (Figure 7c). This trend suggests that the accumulation of interfacial byproducts increasingly promoted competing reactions (e.g., hydrogen evolution), which is a phenomenon commonly observed during inefficient electrodeposition processes.^56^ In sharp contrast, the current-density-time responses of EJSC and the E-bath both appeared relatively moderate (Figure 7b,d), although the underlying mechanisms were fundamentally different. The stability of EJSC stemmed from the forced convection it generated, which efficiently removed deposition byproducts and continuously replenished Ag^+^ ions near the cathode. As a result, concentration polarization at the interface was effectively mitigated,^57^ enabling electroreduction to proceed under a mass-transport-optimized quasi-steady state. In the case of E-bath, the low average current density essentially reflected its limitation by restricted mass transport. The absence of convection further hampered Ag^+^ transport to the cathode, leaving the interfacial concentration persistently depleted. With prolonged electrodeposition, all three strategies exhibited a pronounced decline in efficiency, indicating a mechanistic shift from nucleation-dominated kinetics at the early stage to diffusion-limited growth governed by the gradual depletion of Ag^+^ ions. Compared with EJS and the E-bath, EJSC achieved the highest current efficiency (79.6%) and Ag recovery (92.6%) within 3 min, demonstrating its technological edge in enabling rapid and energy-efficient recovery of secondary resources.

Moreover, differences in mass-transport regimes strongly influence the microscopic morphology of the deposited Ag products. In the EJSC strategy, the dynamically renewed interfacial environment promotes the formation of dense and uniform dendrites with an average grain size of 74.8 nm on the Ag coil surface (Figure 7e). In contrast, the EJS method produces Ag deposits with a mixed morphology and an average grain size of 111.1 nm (Figure 7f), where coarse dendrites coexist with fine single crystals. This phenomenon is consistent with the findings of Zhang et al. in static Ni-JECD,^58^ where continuous ion replenishment at a fixed interface concentrates the local electric field at protrusions, inducing passive vertical growth and irregular dendritic development. However, the E-bath process yields uniform Ag single crystals with an average size of 239.5 nm (Figure 7g). The morphological evolution aligns with the instantaneous nucleation model proposed by Grujicic et al.,^59^ in which mass-transport limitations and slow ion diffusion reduce the interfacial Ag^+^ ion concentration, markedly suppressing nucleation density. Sparse nuclei form only at a limited number of active sites in the early stage, followed by diffusion-controlled lateral growth and grain boundary coalescence, ultimately producing large single-crystal structures. The progression from compact dendrites to sparse single crystals clearly demonstrates the advantage of the actively regulated vertical growth mode in EJSC for enhancing Ag recovery quality, while also highlighting the inherent limitations of passive growth in EJS and the E-bath. The deposited Ag can be readily detached from the cathode as powders, with colors ranging from yellow-gray to metallic-Ag gray, reflecting the intrinsic differences in light response associated with distinct sizes and morphologies. As noted by Sivasubramanian and his colleague,^60^ dense nanodendrites (EJSC), as fractal nanostructures, exhibit selective absorption in the blue-green spectral region, whereas large single crystals (E-bath) approach the optical behavior of bulk Ag, displaying broadband and efficient reflection. Such tunable optical properties, dictated by the recovery pathway, open avenues to valorize the recovered Ag powders in multifunctional applications spanning photocatalysis to highly reflective coatings.^61,62^

Furthermore, we conducted a comprehensive analysis of the crystal phase, purity, and functionality of the Ag powders recovered through three strategies, which revealed the intrinsic connection between their microscopic structures and macroscopic properties. The X-ray diffraction (XRD) patterns demonstrate that all diffraction peaks of the recovered Ag match perfectly with the standard Ag reference (Figure 7h), without any extraneous reflections, confirming the exceptionally high phase purity of the products. The absolute peak intensities are strongest for EJSC, moderate for EJS, and weakest for the E-bath, which is fully consistent with their trend in Ag recovery efficiency. More importantly, the variations in the relative intensities of the crystal planes provide deep insight into how different mass-transfer modes regulate the growth habits of Ag crystals. We observed that as the recovery process shifts from EJSC to the E-bath, the intensity of the Ag(111) reflection gradually decreases relative to the Ag(200) reflection. This trend signifies a change in the dominant growth orientation. Under the forced scanning convection of EJSC, which ensures abundant Ag^+^ supply and continuous nucleation sites, Ag preferentially undergoes rapid epitaxial growth along the close-packed (111) plane, giving rise to vertically oriented dendritic structures. In contrast, under the mass-transfer limitations of the E-bath, the sluggish Ag^+^ supply drives crystal growth toward an isotropic mode, thereby weakening the preferential growth of the (111) plane. At the same time, the progressive inversion of the intensity ratio between the (220) and (311) reflections substantiates the transition of the crystal growth mode from kinetically governed dendritic growth to near-equilibrium growth dominated by diffusion. This decisive role of mass-transfer kinetics in dictating growth behavior resonates with the mechanism reported by Zhang and co-workers, who demonstrated that kinetic factors outweigh thermodynamic equilibrium in controlling island morphology during crystallization.^63^

In addition, the Ag recovered by all three methods reached a purity above 99.4% (Figure 7i), arising from the highly selective dissolution of Ag in dilute HNO_3_ driven by voltage differences during extraction. Complementary compositional analyses (Figure S9 and Table S4) benchmarked the recovered powders against commercial-grade 3N–Ag (99.9%), confirming predominantly Ag with trace impurities, with only minor variations in impurity composition (e.g., Ti, Si, Pb, Bi). Among the three methods, the EJSC process delivered the highest purity of 99.88%, approaching commercial 3N–Ag. This advantage arises from dynamic scanning, which continuously generates fresh nucleation sites, promotes preferential Ag deposition with the most positive reduction potential, and suppresses impurity coreduction. Purity alone, however, is insufficient to define performance as a conductive filler, since microstructure and crystallinity exert equal influence. The magnified Ag(111) diffraction peak (Figure 7h, right) revealed a progressive narrowing of the full width at half-maximum (fwhm) from 0.12° for EJSC dendrites to 0.04° for the E-bath single crystals. According to the Scherrer eq (eq S6), this directly indicates an increase in average crystallite size and aligns with the particle distribution analysis from the SEM micrographs (Figure 7e–g). The broad fwhm of EJSC reflects its nanoscale dendrites composed of numerous small domains, whereas the sharp peak of the E-bath product reflects the highly ordered face-centered cubic (FCC) single crystals.

Electrical measurements (Figure 7j) further highlight that structural distinctions directly govern conductive performance. The EJSC recovered Ag, with its dense dendritic network, rapidly establishes an efficient conductive pathway within the resin through point-line-plane contacts and achieves the lowest bulk resistance at an ultralow loading of 4 mg. The mixed microstructure of EJS yields intermediate performance, while both the E-bath product and commercial 3N–Ag required a higher loading above 6 mg to approach a comparable percolation threshold, likely because their perfect FCC morphology restricts effective intercrystalline contacts and thereby hinders network formation. Consistent with prior reports^64–66^ that dendritic Ag, owing to its fractal geometry and abundant nanoscale tips, markedly lowers the percolation threshold and facilitates efficient conductive network formation at low loading, the EJSC recycling strategy therefore enables direct upcycling of Ag from EoL-SSCs into high-aspect-ratio 3N fillers suitable for advanced device manufacturing (e.g., flexible circuits, electrochemical sensors, and printed electronics).

### 2.7 Industrial Feasibility of EJSC within the PV Recycling Chain

To evaluate the practical relevance of the EJSC process, we position it within the broader landscape of PV module recovery. Figure 8a presents the most mature industrial pathway for decommissioned module treatment, the full recovery end-of-life photovoltaic (FRELP) recycling scheme,^67^ into which EJSC can be seamlessly integrated. The FRELP process begins with the mechanical dismantling (Step 1) of the module’s structural components such as the frame, backsheet, and junction box. The separated laminate is then subjected to delamination (Step 2) to expose the EoL-SSC, recover tempered glass, and remove the ethylene–vinyl acetate (EVA) encapsulant. In practical industrial deployment, thermal delamination has become increasingly preferred because the EVA layer undergoes clean thermal decomposition that enables nearly complete separation, whereas solvent-based delamination remains limited by waste generation and low throughput.^68^ In conventional FRELP practice,^67^ the detached EoL-SSC is normally comminuted together with glass, backsheet fragments, and residual metallic components.^3^ The resulting mixture is sieved to obtain a homogeneous particulate fraction with sizes of several hundred micrometers. Although this physical pretreatment increases the specific surface area and improves the leaching kinetics of Ag, it also causes extensive codissolution of base metals (e.g., Al, Cu and Pb). Such an impurity-rich leachate necessitates multiple purification steps to achieve selective Ag recovery. Notably, EJSC recycling (Step 3) provides a fundamentally different approach from conventional acid leaching routes. It removes Ag directly from the intact EoL-SSC and produces high-purity Ag through a single-step electrodeposition process (Step 4). Because the functional integrity of the PV substrate is preserved, the process also establishes a technically viable basis for subsequent refurbishment. Overall, replacing chemical-intensive metal recovery with EJSC at the final FRELP stage eliminates the requirement for material classification and wafer etching,^47^ while offering a promising opportunity to further reduce manufacturing costs and environmental burdens across the PV value chain.

**Figure 8:**
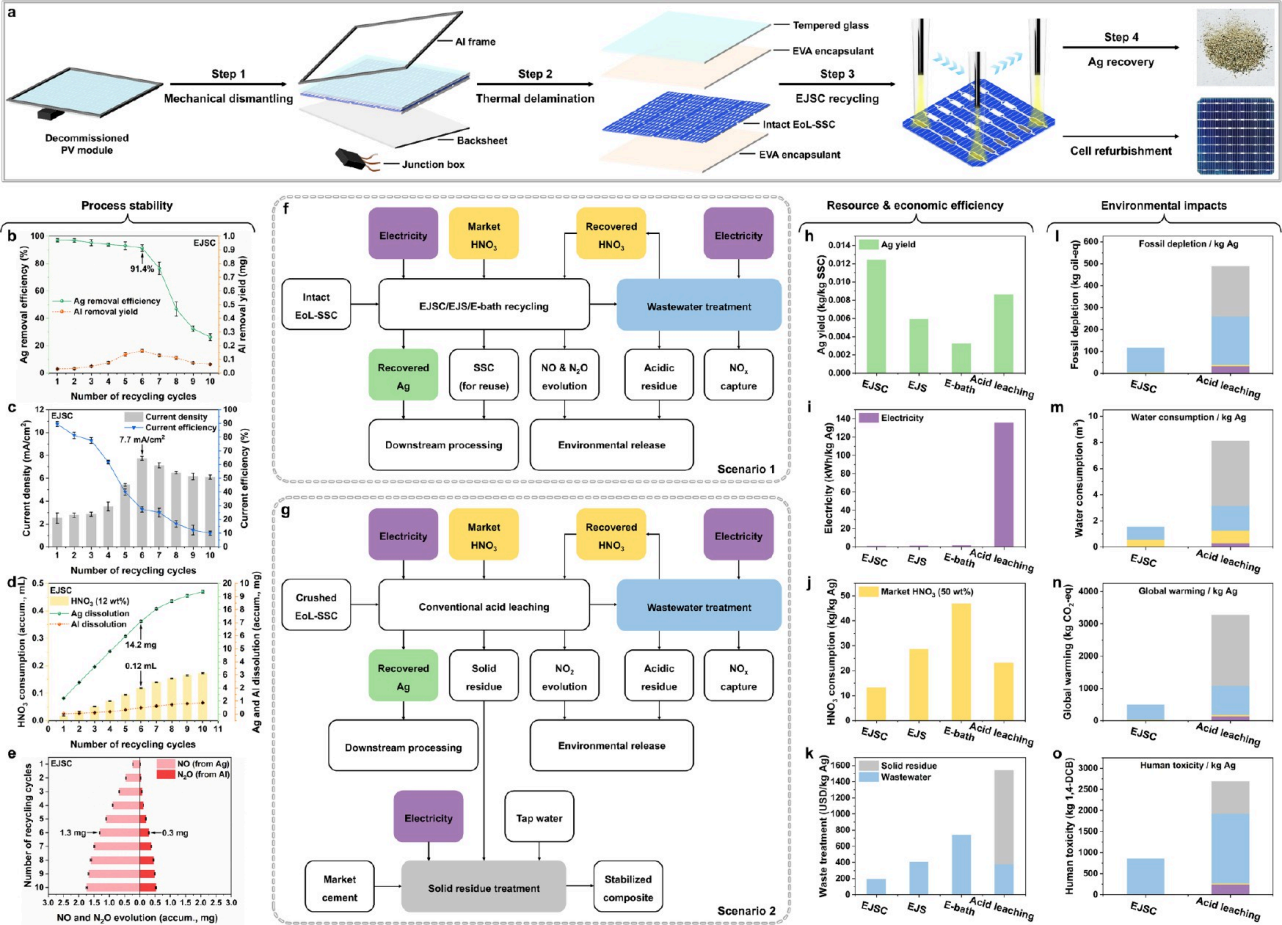
Industrial feasibility and integrated LCA–TEA assessment highlighting the multidimensional advantages of EJSC over alternative Ag recovery routes within the PV recycling chain. (a) Schematic illustration of the FRELP recycling workflow with EJSC integration. Process stability: (b) ICP-MS quantification of Ag removal efficiency and Al dissolution yield across ten sequential EJSC runs using fresh EoL-SSCs in an unreplenished 12 wt % HNO_3_ reservoir. (c) Evolution of average current density and Ag-specific current efficiency. (d) Accumulated Ag and Al dissolution with corresponding 12 wt % HNO_3_ consumption. (e) Cumulative NO and N_2_O evolution, highlighting NO as the dominant gaseous product. Life-cycle scenarios: (f, g) Process flowsheets for the electrochemical route in 12 wt % HNO_3_ (Scenario 1) and the conventional acid-leaching route in 25 wt % HNO_3_ (Scenario 2). Resource and economic efficiency: (h) Ag yield across different recovery methods per 1 kg of SSC. (i–k) Electricity use, market HNO_3_ consumption, and waste treatment cost per kg of recovered Ag. Environmental impacts: (l–o) Core impact categories contrasting the EJSC and acid-leaching routes, including fossil depletion, water consumption, global warming, and human toxicity associated with recovering 1 kg of Ag. Error bars, where applicable, represent ±1 SD; *n* = 3.

To emulate continuous industrial operation, we subjected independent EoL-SSC samples to successive treatments in 30 mL of unreplenished 12 wt % HNO_3_, enabling a direct evaluation of the durability of the EJSC process. ICP-MS analysis (Figure 8b) indicates that the Ag removal efficiency remained above 91.4% during the first six cycles, whereas the dissolved Al increased from 0.03 mg to 0.16 mg and subsequently declined as the oxidizing strength of the electrolyte weakened with nitrate depletion. Although lower nitrate concentrations generally suppress Al corrosion, previous studies have shown that trace metal impurities such as Cu^2+^ can accelerate electron transfer between Al and nitrate,^69^ which provides a mechanistic rationale for the activated Al dissolution observed in the early stages. Electrochemical measurements (Figure 8c) reinforce this interpretation. The current efficiency decreases from an initial 89.6 to 27.5% in the sixth cycle, while the current density rises to a peak of 7.7 mA/cm^2^ in the same cycle before declining to a steady plateau. In comparison, the EJS process (Figure S10a) sustains stable Ag removal for only three cycles, and the E-bath method (Figure S10e) begins to deteriorate immediately. Both approaches undergo a rapid increase in Al dissolution accompanied by a progressively rising current density (Figure S10b,f), reflecting a growing contribution from impurity-driven parasitic reactions. These findings demonstrate that EJSC exhibits markedly enhanced kinetic stability during consecutive SSC processing cycles, sustaining high Ag extraction performance for up to six cycles.

From the perspective of material flows, we further quantified the cumulative metal dissolution, while the associated acid consumption and gaseous byproduct formation were determined from the stoichiometric relationships governing Ag and Al dissolution (eqs S7–S12b). The stoichiometric estimation (Figure 8d) indicates that EJSC dissolves 14.2 mg of Ag within the first six cycles while requiring only 0.12 mL of 12 wt % HNO_3_, and the acid consumption remains tightly correlated with the progression of Ag dissolution. Even after ten consecutive treatments, the accumulated impurities represented by Al remain below 1 mg, and the total acid consumption corresponds to merely ∼0.6% of the initial 30 mL reservoir, suggesting that electrolyte degradation and recirculation continuity are minimally affected. The gaseous products generated during Ag and Al dissolution (Figure 8e) consist primarily of 1.3 mg low-toxicity NO and 0.3 mg inert N_2_O, rather than nitrogen dioxide NO_2_, a highly reactive atmospheric pollutant typically generated in concentrated acid leaching routes.^8^ In contrast, both EJS and E-bath exhibit a pronounced escalation in acid consumption as Al dissolution accelerates (Figure S10c,g), and the relationship between acid usage and Ag recovery becomes increasingly decoupled. The accumulated Al in these two systems reaches 2–4 times that of EJSC, yielding substantially higher N_2_O release relative to NO (Figure S10d,h). The effect is particularly evident for EJS, which generates 1.1 mg of N_2_O within the first six cycles and rises to 2.8 mg after ten samples, revealing an accelerated amplification that may intensify greenhouse gas (GHG) forcing and stratospheric ozone depletion^70^ during scale-up operations. Taken together, these results confirm that EJSC achieves superior electrolyte utilization and more effective mitigation of waste emissions during batch processing of EoL-SSCs, underscoring its strong potential for sustainable and industrially scalable deployment.

Beyond the conventional crystalline-Si architectures, such as the passivated emitter and rear cell (PERC) examined in this work, the EJSC strategy can be readily extended to next-generation SSCs, particularly silicon heterojunction (SHJ) and tunnel-oxide passivated contact (TOPCon) devices.^71,72^ Both technologies retain a front-side metallization stack in which screen-printed Ag grids sit on a transparent conductive oxide (TCO) above ultrathin passivation layers. This shared Ag–TCO–passivation sequence renders the Ag grid the only electrochemically labile component. Although the passivation layer in SHJ cells is hydrogenated amorphous silicon (a-Si:H), whereas in TOPCon cells it is a SiO_2_/poly-Si stack, both layers exhibit electronic properties that are highly sensitive to hydrogen redistribution, dopant activation, and local field perturbations, underscoring the importance of limiting electrochemical exposure. Unlike conventional acid leaching and E-bath approaches, which expose the entire device stack to aggressive chemistries, the spatial and dynamic advantages of EJSC can focus the applied field on Ag and restrict electrolyte residence to the metal–TCO interface, thereby mitigating risks associated with prolonged or uncontrolled interfacial reactions. Moreover, SHJ and TOPCon cells typically lack the front-side Al metallization present in PERC, which simplifies selectivity control, broadens the accessible potential window for Ag dissolution, and suppresses Al codissolution as well as the associated GHG emissions. Overall, these considerations indicate that EJSC is not only broadly compatible with advanced high-efficiency PV technologies but also provides a more environmentally benign pathway to Ag recovery.

### 2.8 Integrated Life-Cycle and Technoeconomic Assessment

A comprehensive life cycle assessment (LCA) was conducted to characterize the economic, resource, and environmental performance of the three electrochemical strategies relative to conventional acid leaching. The study followed the ISO 14040/14044:2006 framework, adopted 1 kg of EoL-SSCs as the functional unit (FU), and integrated experimentally derived data with the ecoinvent 3.9.1 database. Scenario 1 (Figure 8f) represents the electrochemical route and was parametrized using the stability-verified operating conditions obtained across six consecutive EJSC, EJS, and E-bath recycling cycles. This scenario captures the characteristic electricity demand, market HNO_3_ (50 wt %) consumption, and gaseous nitrogen evolution intrinsic to the active and passive electrochemical dissolution of Ag and Al-based impurities. Scenario 2 (Figure 8g) represents the conventional hydrometallurgical route and was constructed from the high-efficiency acid-leaching conditions reported by Song et al.,^8^ in which a continuous stirred-tank reactor achieved 62% Ag recovery within 300 s. This scenario reflects a mainstream industrial practice involving crushed EoL-SSC feedstock, nonreusable solid residues, and NO_2_-dominated off-gas emissions. Given that downstream sectors such as fine chemical and metallurgical processing require diverse commercially usable Ag products (e.g., AgNO_3_, Ag_2_O, or metallic Ag), the system boundaries for both scenarios were uniformly defined at the point of recovering Ag^+^ as the intermediate product. To precisely assess the closed-loop potential of the core recycling processes, wastewater treatment was modeled using a chemical-free vacuum-distillation system,^73^ in which HNO_3_ and water are separated through evaporation–condensation and the recovered acid is internally recirculated. This configuration avoids secondary emissions associated with external chemical inputs, and any NO_*x*_ generated during treatment is assumed to be fully absorbed within the distillation unit and therefore treated as having zero direct emissions in the elementary flow. Another key distinction between the two scenarios concerns the management and fate of solid products. The electrochemical route yields an intact SSC suitable for direct reuse, whereas the conventional acid-leaching route generates solid residues that require cement-based stabilization to mitigate heavy-metal burdens.^74^ Collectively, this LCA model establishes a coherent pathway that reflects established industrial reality and aligns with future sustainability trajectories.

The inventory data for both Scenarios were normalized using experimentally measured values and literature derived parameters (Tables S5–S8), and the acid consumption and NO_2_ evolution in Scenario 2 were obtained from stoichiometric conversions for 25 wt % HNO_3_ (∼5 M; eqs S13a–S14b). The complete EoL-SSC sheet used in this work weighed 10.3 g and contained 54 Ag pads with a total Ag mass of 0.135 g, corresponding to an original Ag content of 1.31%. This value is very close to the 1.39% Ag content reported for crushed SSC particles,^8^ providing a reliable basis for comparing the two Scenarios. As an illustrative case in Scenario 1, the inventory data for the EJSC process are derived using a stock solution volume of 30 mL together with the average Ag removal efficiency and current density from the first six cycles (Figure 8b,c). These inputs lead to an overall acid requirement of 6.73 kg and an electricity use of 0.012 kWh per FU, yielding ∼0.012 kg of Ag and ∼0.75 kg of secondary PV cells (Table S5). Although the EJS and E-bath processes operate with lower electricity inputs (Tables S6 and S7), their Ag yields of ∼0.006 kg and ∼0.003 kg remain substantially lower than that of EJSC (Figure 8h). The acid leaching process in Scenario 2 yields ∼0.009 kg of Ag, the second highest output among all processes, under high speed stirring at 800 rpm and heating at 40 °C.^8^ Nevertheless, its electricity demand for Ag recovery reaches 1.2 kWh per FU (Table S8), nearly 100 times that of EJSC.

When expressed per kg of recovered Ag (Figure 8i), the EJSC route requires only 0.94 kWh of electricity, whereas acid leaching reaches 135.5 kWh, the highest among all evaluated pathways. This substantial burden stems from the initial acid reserve of 800 mL per 50 g of SSC,^8^ which generates a significantly larger wastewater volume and necessitates additional energy for stabilizing the solid residues. Since vacuum distillation can recover 98% of HNO_3_,^73^ the actual acid consumption of each process is therefore dictated mainly by the stoichiometric amount per unit mass of Ag recovered. Consequently, the market HNO_3_ (50 wt %) demand of the E-bath process becomes extremely high when normalized to its very limited Ag output, in contrast, EJSC maintains the lowest requirement at 13.2 kg/(kg of Ag) (Figure 8j). From an integrated waste-management perspective (Figure 8k), although the cost of treating spent electrolyte increases from the dynamic EJSC process to the passive E-bath route, both values remain less than half of the disposal expense associated with acid-leaching waste. In the latter case, more than 75% of the total cost arises from solid-residue treatment, which consumes supplementary purchased resources such as cement and tap water.^74^

The techno-economic assessment (TEA) further provides a quantitative comparison across the two scenarios (Table 1). EJSC delivers the lowest net process cost at 211.5 USD/kg Ag, amounting to less than one-seventh of the acid-leaching expense (1673.1 USD/(kg of Ag)). Using the 2026 spot Ag price (∼2400 USD/kg), EJSC yields the highest net Ag profit (2188.5 USD/(kg of Ag)), substantially surpassing both EJS and the E-bath process. Notably, EJSC is the only strategy that generates added value through the retention of a reusable PV substrate, while EJS and the E-bath route may require a postetching step^47^ to fully eliminate residual Ag. Based on the market price of solar-grade Si (6.86 USD/kg),^75^ the substrate contributes an additional 414.2 USD/(kg of Ag), enabling a potential total revenue exceeding 2500 USD/(kg of Ag) at commercial scale. Viewed holistically, these integrated LCA–TEA indicators demonstrate that EJSC not only reduces resource dependence but also maximizes secondary value creation, establishing a strong economic driver for future integration into PV recycling infrastructure.

**Table 1:** Technoeconomic Comparison of Ag Recovery Routes under Different Life Cycle Scenarios

life cycle scenario	recycling method	net process cost (USD/(kg of Ag))	net Ag profit (USD/(kg of Ag))	substrate added value (USD/(kg of Ag))
Scenario 1	EJSC	211.5	2188.5	414.2
	EJS	442.2	1957.8	
	E-bath	805.4	1594.6	
Scenario 2	acid leaching	1673.1	726.9	

To systematically evaluate the environmental sustainability of the different Ag recovery pathways, all impact categories were quantified using the ReCiPe 2016 Midpoint (H) method (Table S9). The results indicate that EJSC exhibits pronounced advantages in both resource consumption and emission generation. Its fossil resource scarcity (116.04 kg of oil-equiv/(kg of Ag)) is only about half that for EJS and roughly one-quarter for acid leaching, reflecting the benefits of dynamic electrolyte renewal and localized reaction zones in reducing process energy demand. Water consumption for EJSC (1.53 m^3^/(kg of Ag)) is also substantially lower than that of the other pathways, especially when compared with acid leaching (8.09 m^3^/(kg of Ag)). This reduction arises from electrolyte recirculation, which significantly decreases solvent replenishment and wastewater discharge. Industrial HNO_3_ production is an energy-intensive upstream process associated with GHG emissions dominated by N_2_O, whose global warming potential is 265–298 times higher than that of CO_2_,^76^ making it a key contributor to the carbon footprint of Ag recovery. Owing to its minimal acid requirement and the predominance of NO rather than N_2_O in the resulting emissions (Figure 8d,e), EJSC achieves a global warming potential (485.9 kg of CO_2_-equiv/(kg of Ag)) that is reduced by ∼85% relative to conventional acid leaching. EJSC also registers the lowest burdens in toxicity-related categories, including human carcinogenic toxicity (41.74 kg of 1,4-DCB/(kg of Ag)) and human noncarcinogenic toxicity (805.8 kg of 1,4-DCB/(kg of Ag)). These reductions stem from its high Ag selectivity, which suppresses stray corrosion and limits the release of heavy metal ions (e.g., Pb, Bi, Cu, and Zn) into the liquid phase. EJSC further outperforms the other pathways in freshwater, marine, and terrestrial ecotoxicity, demonstrating a higher degree of environmental compatibility at the ecosystem scale.

A process-level contribution analysis further separates the influences of electricity use, HNO_3_ consumption and waste management on the major impact categories, clarifying the origins of EJSC’s advantages over acid leaching. For fossil depletion (Figure 8l), the dominant contribution in EJSC arises from wastewater treatment because vacuum distillation requires thermal input to achieve HNO_3_–H_2_O separation.^73^ In acid leaching, fossil fuel demand is instead driven by upstream HNO_3_ production and transportation. For water consumption (Figure 8m), EJSC is influenced by HNO_3_ use and by the nonrecyclable water lost during distillation. Acid leaching shows much higher freshwater demand due to the large volumes of spent liquor and solid residues that must be processed. For global warming potential (Figure 8n) and human toxicity (Figure 8o), EJSC is influenced simultaneously by wastewater treatment and by the subsequent release of acidic residues. Acid leaching, however, exhibits a different pattern. Its GHG emissions are dominated by upstream cement production,^74^ which accounts for ∼8% of global CO_2_ emissions,^77^ while its human-toxicity impact is driven by the metal-rich residues formed during the low-selectivity dissolution step.

Moreover, across media-spanning impact categories such as fine particulate matter formation, ionizing radiation, and land use (Table S9), EJSC consistently yields the lowest environmental burdens. This pattern indicates that its process design avoids shifting environmental loads across different compartments and instead enables coordinated reductions in emissions. Notably, the E-bath exhibits higher impacts than conventional acid leaching in human toxicity, aquatic ecotoxicity, and stratospheric ozone depletion. This contrast reflects the fundamentally different pollution pathways of electrochemical and chemical dissolution. The burdens of the electrochemical route are driven by continuous electricity demand, which is indirectly associated with heavy-metal emissions and halogenated ozone-depleting species^78^ through a fossil-dominated power grid. In comparison, the chemical route is dominated by the energy-intensive production and regeneration of HNO_3_. From a life-cycle perspective, EJSC demonstrates a clear environmental advantage in the high-value recovery of PV waste. Further improvements may be achieved by reducing reliance on HNO_3_ through greener media (e.g., DES and MSA)^10,18^ or by integrating sustainable energy sources (e.g., nuclear power and hydropower)^79,80^ into upstream electricity supply. Such developments would help advance PV-based urban-mining practices aligned with carbon neutrality and resource circularity.

## 3 Conclusions

In this work, we demonstrate EJSC as an efficient and selective “top-down” recycling strategy for EoL-SSCs, enabling rapid Ag extraction (97.1% in 4 min) under mild conditions while preserving the underlying PV substrate for potential reuse. Confined microreaction control and continuous interfacial renewal overcome the mass-transport limitations of static or passive systems, achieving uniform, layer-by-layer Ag removal. The dissolved Ag^+^ ions are directly upcycled into 3N-purity (99.88%) dendritic Ag powders with high recovery efficiency (92.6% in 3 min), forming conductive networks suitable for advanced electronic applications. EJSC also exhibits pronounced process stability and strong industrial feasibility, maintaining high Ag-removal performance (>91%) over six treatments, with minimal Al dissolution and exceptionally low acid consumption even after ten consecutive cycles. LCA–TEA analyses further show that this operational durability translates into substantially lower environmental burdens and improved economic performance compared with conventional acid-leaching routes, reflected by an ∼85% reduction in global warming potential and yielding a revenue more than 3-fold higher per kg of Ag recovered. The confined-jet principle additionally suggests native compatibility with the thin-film passivating contacts and delicate surface passivation stacks of emerging PV technologies such as SHJ and TOPCon, indicating broader applicability beyond conventional Si cells. This work positions EJSC as a high-efficiency, digitally orchestrated electrochemical platform that delivers economically viable and environmentally benign recovery of critical metals, establishing a closed-loop pathway for PV waste upcycling within the future landscape of sustainable urban mining.

## Supplementary Material



## Data Availability

All data supporting the findings of this study are available within the article and the Supporting Information file, or available from the corresponding authors upon request.

## Notes

Experimental details and stoichiometry and LCA methodology along with supplementary figures and tables (PDF)
