# Supplementary material for: Continuous Electrolyte Jet Scanning Enabled Near-Quantitative and High-Purity Silver Recovery from Silicon Solar Cells
Source: JACS Au. 2026 Feb 19;6(3):1625–42. doi: 10.1021/jacsau.5c01469 (PMC13014220; doi:10.1021/jacsau.5c01469)
Supplement: Supplementary file 1 [file au5c01469_si_001.pdf]

Supporting Information

Continuous Electrolyte Jet Scanning Enabled Near-Quantitative and High-Purity Silver Recovery from Silicon Solar Cells

Wending Gu*, David Payne, Shujuan Huang, and Binesh Puthen Veettil*

School of Engineering, Macquarie University, Sydney, 2109 NSW, Australia

*Corresponding authors:

W.G., wending.gu@hdr.mq.edu.au

B.P.V., binesh.puthenveettil@mq.edu.au

Table of Contents

1. EXPERIMENTAL SECTION 4

1.1. Materials and Sample Preparation 4

1.2. Electrolyte Formulation and Benchmark Leaching 4

1.3. Electrochemical Recycling System Setup 4

1.4. Electrochemical Measurements and Analysis 5

1.5. ICP-MS Quantification of Recycled Electrolytes 7

1.6. Microstructural, Compositional, and Electrical Characterization 7

2. STOICHIOMETRY AND LCA METHODOLOGY 10

2.1. Reaction Stoichiometries of Metal Dissolutions in Dilute and Concentrated HNO_3 10

2.2. Stoichiometric Conversion to HNO_3 Consumption and Gaseous Nitrogen Emissions 11

2.3. Uncertainty Propagation 12

2.4. LCA Methodology 12

3. SUPPLEMENTARY FIGURES AND TABLES 13

Figure S1 13

Table S1 14

Figure S2 15

Table S2 16

Figure S3 17

Figure S4 18

Figure S5 19

Figure S6 20

Figure S7 21

Table S3 22

Figure S8 23

Figure S9 24

Table S4 25

Figure S10 26

Table S5 27

Table S6 28

Table S7 29

Table S8 30

Table S9 31

REFERENCES 32

1. EXPERIMENTAL SECTION

1.1. Materials and Sample Preparation

Rectangular end-of-life silicon solar cell (EoL-SSC) samples (2.7 × 1.5 cm) were sectioned from retired monocrystalline passivated emitter and rear cells (PERC) modules (9-busbar design, Zoeast PV, China) using a laser cutter (xTool F1 Ultra, China). Samples were sequentially ultrasonicated in ethanol (100%, Chem-Supply Pty Ltd., Australia) and deionized water (Milli-Q, Merck Millipore, Germany) for 5 min each, followed by drying under vacuum at 25 °C. To define a reproducible geometric area of 4 cm^2, the rear contacts were insulated with electrical insulation tape (3M™ Scotch, USA), leaving only the Ag pads, Al contacts, and the front-side cell architecture exposed.

1.2. Electrolyte Formulation and Benchmark Leaching

A 12 wt % nitric acid (HNO_3) electrolyte for electrolyte jet scanning (EJSC), electrolyte jet static (EJS), and electrolytic bath (E-bath) recycling was prepared by diluting 20 g of concentrated HNO_3 (70 wt %, AR grade, Westlab Pty. Ltd., Australia) in 100 mL of Milli-Q water. To quantify the total Ag content and establish a realistic benchmark for hydrometallurgical performance, two complementary acid leaching experiments were conducted. For total Ag quantification, separate EoL-SSC specimens with the rear side insulated were immersed in concentrated HNO_3 (70 wt%) and statically leached for 48 h to achieve complete dissolution and provide the reference Ag inventory. For benchmarking, a 5 M HNO_3 solution was prepared by diluting 31.6 mL of 70 wt% HNO_3 with Milli-Q water to a final volume of 100 mL. Intact SSCs were immersed in this 5 M HNO_3 at 25 °C with stirring at 200 rpm for 1 h, using a magnetic stirrer (IKA, Germany). This condition was selected from representative literature reports[1-6] and serves as a laboratory-validated, industrially relevant benchmark for conventional acid leaching.

1.3. Electrochemical Recycling System Setup

The electrolyte jet (EJ) recycling system was operated in both EJSC and EJS modes using a previously reported electrochemical 3D printer design (Figure S1).[7, 8] The printer was based on a commercial three-axis motion stage (Felix 3.0, Netherlands). The single-screw extruder was replaced with an electrochemical nozzle assembly comprising a 1 mL luer slip polypropylene syringe (Terumo, Japan) fitted with a micropipette tip (Livingstone, Australia; inner diameter 2 mm). EoL-SSC samples were mounted on an electrochemical platform comprising a 3D-printed acrylonitrile butadiene styrene (ABS) hollow stage placed in a 100 mL glass beaker and electrically connected as the working electrode (WE). A carbon fiber rod (Goodfellow, UK; diameter 1 mm) was inserted through the nozzle body, sealed with a rubber septum, and extended vertically to the nozzle tip, serving as the combined counter and reference electrode (CE/RE). This integrated quasi-reference electrode configuration minimized variations in

solution resistance during nozzle movement and maintained stable potential control at the Ag dissolution site, while avoiding metallic ion leakage associated with conventional AgCl electrode.[9, 10]

The interelectrode gap (IEG), defined as the distance between the carbon fiber cathode and the Ag pad surface, was systematically varied from 0.5 to 6.5 mm in 2 mm increments to assess its effect on current density and electric field distribution. Electrolyte circulation was maintained by a custom two-channel peristaltic pump that continuously recirculated a 30 mL reservoir of diluted HNO_3 (12 wt %) at 240 mL/min, which represented the maximum flow rate without turbulence and maintained a confined reaction microzone on the Ag pads. The electrolyte was delivered through Teflon tubing (inner diameter 4 mm). The nozzle was mounted on the X axis belt using a 3D-printed ABS holder and translated laterally across the Ag pads at programmed scanning velocities of 6-9 mm/s (G-code control). Prior to each experiment, the nozzle-substrate distance (Z-offset) was calibrated to 0.5 mm using a numerical controller. This setup established a confined micro-reaction zone on the Ag pad, minimized nozzle clogging, and suppressed stray current corrosion. For comparison, bulk electrolysis recycling was performed in a commercial E-bath (Ossila, UK). The dual-electrode configuration was retained, with the carbon fiber rod and EoL-SSC arranged in parallel at the optimal IEG of 4.5 mm determined from the EJSC experiments, immersed in 30 mL of 12 wt % HNO_3.

1.4. Electrochemical Measurements and Analysis

A potentiostat (Ossila, UK) was employed to control the voltage/potential of the EoL-SSC WE relative to the carbon fiber CE/RE. Initial characterization was performed by linear sweep voltammetry (LSV) at a scan rate of 10 mV/s to evaluate the current density (normalized to the geometric area) as a function of applied voltage (0.0-4.0 V), with the nozzle positioned over either the Ag electrode or the Al busbar region. From these measurements, 2.0 V was identified as the optimal voltage for selective anodic dissolution of Ag in the dual-electrode recycling system. The open-circuit potential (OCP) of the carbon fiber rod was measured at 25 °C in saturated KCl solution (≥ 99.0%, Sigma-Aldrich, Australia) against a platinum wire CE (99.99%, Sigma-Aldrich; 0.5 mm diameter) and an Ag/AgCl reference electrode (Arca Technology Store, China; 4 mm diameter). The measured OCP was -0.8183 V. Considering the standard potential of Ag/AgCl relative to the standard hydrogen electrode (SHE, +0.197 V), the potential of the carbon fiber rod was determined to be -0.6213 V vs. SHE. All subsequent potentials are reported on this scale.

To investigate electrochemical kinetics, LSV was first performed between -2.0 and 0 V (vs. SHE) at 10 mV/s to identify the non-Faradaic region. Within this window, cyclic voltammetry (CV) was conducted at 20 mV/s for 50 cycles to stabilize the EoL-SSC/electrolyte interface. Subsequently, CVs were recorded at scan rates from 10 to 50 mV/s in 10 mV increments, yielding quasi-rectangular profiles. The double-layer capacitance (Cdl) was determined from

the slope of the average current density versus scan rate in the non-Faradaic region. The electrochemically active surface area (ECSA) was estimated using a representative specific capacitance (Cs) of 40 μF/cm^2, intermediate between values typically reported for pure metals (20 μF/cm^2) and metal oxides (60 μF/cm^2).[11] Finally, LSV was carried out between 0 and 1.6 V (vs. SHE) at 10 mV/s to systematically evaluate the effects of IEG, nozzle scanning velocity, HNO_3 concentration, and Ag extraction strategy on the ECSA-normalized current density. Tafel slopes were derived from linear fitting of the logarithm of the specific current density versus overpotential, enabling quantitative evaluation of the kinetics and identification of the rate-determining steps in Ag dissolution.

Chronoamperometry was employed to assess the energy efficiency of Ag extraction and recovery by monitoring the anodic and cathodic current response as a function of time. During the anodic dissolution step, metallic Ag in the EoL-SSC sample was oxidized to release Ag^+ into solution, with oxygen evolution (OER) as the principal competing process. In the subsequent cathodic reduction step, Ag^+ ions were reduced onto an Ag coil (99.9%, Sigma-Aldrich; 1 mm diameter), yielding Ag nanoparticles, while hydrogen evolution (HER) occurred as a parasitic side reaction. The overall electrochemical reactions are summarized below.

Dissolution of Ag:

$$\mathrm{Ag} \rightarrow \mathrm{Ag^+} + \mathrm{e^-}\ (\mathrm{Extraction});\ 2\mathrm{H_2O} \rightarrow \mathrm{O_2} + 4\mathrm{H^+} + 4\mathrm{e^-} (\mathrm{OER})$$

Reduction of Ag:

$$\mathrm{Ag^+} + \mathrm{e^-} \rightarrow \mathrm{Ag}\ (\mathrm{Recovery});\ 2\mathrm{H^+} + 2\mathrm{e^-} \rightarrow \mathrm{H_2} (\mathrm{HER})$$

Because parasitic side reactions consume part of the applied charge, the fraction of current available for Ag dissolution and deposition is reduced, thereby lowering the overall current efficiency. To quantitatively assess the selectivity of the Ag reaction, the experimental Ag mass ($m_{\mathrm{Ag,exp}}$) in the recycled electrolytes, as determined by inductively coupled plasma mass spectrometry (ICP-MS, iCAP MSX, Thermo Scientific, Germany), was compared with the theoretical Ag mass ($m_{\mathrm{Ag,theo}}$) calculated from Faraday's law. The Ag-specific current efficiency (η_{Ag}) was obtained according to Eqs. S1 and S2:

$$\eta_{\mathrm{Ag}}(\%) = \frac{m_{\mathrm{Ag,exp}}}{m_{\mathrm{Ag,theo}}} \times 100 \quad \text{(S1)}$$

$$m_{\mathrm{Ag,theo}} = \frac{I \times t \times M}{n \times F} \quad \text{(S2)}$$

where I is the applied WE current (A), t is the reaction time (s), M is the molar mass of Ag (107.87 g/mol), n is the number of electrons transferred per Ag atom (1), and F is the Faraday constant (96485 C/mol).

1.5. ICP-MS Quantification of Recycled Electrolytes

Quantitative analysis of Ag and co-dissolved metal impurities (Al, Ti, Bi, Pb, Cu, Mn, Zn) in the recovered electrolytes was performed by ICP-MS. Calibration curves were generated by external standardization using a certified multi-element stock solution (Multi Element IV, Merck Life Science, Germany). Standard concentrations of 0, 1, 10, 50, 100, and 250 mg/L (ppm) were prepared in 2 wt% HNO_3 in 10 mL polypropylene screw-cap tubes (Sarstedt, Germany) to suppress hydrolysis and minimize adsorption to container walls. Electrolyte samples obtained from electrochemical recycling (EJSC, EJS, and E-bath) and benchmark acid leaching in 5 M HNO_3 for 1 h (200 rpm) were diluted 250-fold with 2 wt% HNO_3 to minimize matrix interferences prior to ICP-MS analysis and ensure accurate quantification. In contrast, leachates generated by Ag reference acid leaching in 70 wt% HNO_3 for 48 h were diluted 500-fold with 2 wt% HNO_3 to determine the maximum recoverable Ag and co-dissolved impurities, as summarized in Table S1.

The mass of each element (m_i) was calculated from its measured concentration (C_i) and the total electrolyte volume (0.03 L) according to Eq. S3.

$$m_i(\mathrm{mg}) = \frac{C_i\left(\frac{\mu\mathrm{g}}{\mathrm{L}}\right) \times 0.03\ (\mathrm{L})}{1000} \tag{S3}$$

The Ag removal efficiency (E_{Ag}) was determined as the ratio of the actual Ag removal mass ($m_{\mathrm{Ag,removal}}$) to the total Ag mass ($m_{\mathrm{Ag,total}}$) obtained from Ag reference acid leaching (Table S1), as expressed by Eq. S4.

$$E_{\mathrm{Ag}}(\%) = \frac{m_{\mathrm{Ag,removal}}}{m_{\mathrm{Ag,total}}} \times 100 \tag{S4}$$

The Ag recovery efficiency (R_{Ag}) was calculated as one minus the ratio of the residual Ag mass in the recycled electrolyte ($m_{\mathrm{Ag,residual}}$) to the Ag mass removed under the optimal EJSC condition at 4 min ($m_{\mathrm{Ag,removal}}$), as defined in Eq. S5.

$$R_{\mathrm{Ag}}(\%) = (1 - \frac{m_{\mathrm{Ag,residual}}}{m_{\mathrm{Ag,removal}}}) \times 100 \tag{S5}$$

1.6. Microstructural, Compositional, and Electrical Characterization

The microstructural features of the Ag pad and Al busbar after electrochemical recycling were examined using field emission scanning electron microscopy (FESEM, JSM-7100, JEOL Ltd., Japan). Imaging was performed at an accelerating voltage of 15 kV and a working distance of 10 mm. Elemental composition analysis of selected regions including the center and edge of the Ag pad and the outer Al busbar was conducted using the energy-dispersive X-ray spectroscopy (EDS) detector integrated with the FESEM. The EDS measurements were

acquired with a live time of 30 s per line, and elemental quantification was performed using atomic number, absorption, and fluorescence (ZAF) correction. This procedure enabled quantitative determination of the residual distribution of Ag and Al as well as other elements originating from underlying cell components such as Ti (from the antireflection coating), Bi (from the glass frit), and Si (from the crystalline wafer substrate).

Micro X-ray fluorescence (XRF) spectroscopy (M4 Tornado, Bruker, Germany) was employed to obtain 2D scans across the entire Ag pad. Measurements were conducted under vacuum conditions at 50 kV and 600 μA. The scan area corresponded to the full Ag pad (~7.7 mm^2), with a spatial step size of 50 μm, enabling assessment of the macroscopic uniformity and extent of Ag dissolution. To complement the XRF results, large-area EDS (Phenom XL, Thermo Fisher Scientific, USA) was performed to evaluate the residual distribution of metallic elements under optimized conditions. The large-area EDS mapping was conducted at 15 kV with a magnification of 1000×. This analysis provided spatially resolved information on trace elements, including manganese (Mn, from the hole transport layer), copper (Cu, used as a conductive additive), zinc (Zn, used as a conductive additive), and lead (Pb, present as a glass modifier). Photovoltaic performance of pristine, EJSC-treated, and acid-leached SSCs were measured using a source measure unit (SMU-4001, UK) under simulated Air Mass 1.5 Global illumination (irradiance of 100 mW/cm^2) provided by a calibrated solar simulator (ORIEL Sol3A, USA). Current–voltage (I–V) curves were obtained by sweeping the applied bias from –1.0 to +1.0 V, thereby enabling a comparative assessment of device efficiency, hysteresis phenomena, and interfacial passivation quality in these recycled samples. Together, the integrated datasets from XRF, macroscopic EDS, and photovoltaic characterization enabled a comprehensive evaluation of the reusability and recyclability of the EoL-SSC after Ag extraction, thereby highlighting the unique advantages of the EJSC strategy.

The crystal phase and preferred orientation of the recovered Ag powders were characterized by X-ray diffraction (XRD, Panalytical Aeris, Netherlands) with Cu Kα radiation (λ = 1.5406 Å). Diffraction patterns were collected over a 2θ range of 30°-90° with a step size of 0.02° and a scan rate of 0.5°/min. All reflections were indexed to the face-centered cubic (FCC) structure of metallic Ag (JCPDS No. 04-0783), with the most intense reflections at 38.1° (111), 44.3° (200), 64.5° (220), 77.4° (311), and 81.5° (222). Relative reflection intensities were compared to assess preferred orientation under different recovery methods. The average grain size was estimated from the full width at half-maximum (FWHM) of the Ag (111) reflection using the Scherrer equation (Eq. S6):

$$D = \frac{K \times \lambda}{\beta \times cos\ \theta} \tag{S6}$$

where D is the grain size, K is the shape factor (0.94), λ is the X-ray wavelength, β is the FWHM (in radians), and θ is the Bragg angle. The calculated grain sizes were compared with the particle size distribution obtained from FESEM analysis.

The purity of recovered Ag, benchmarked against commercial 3N-Ag (99.9%, Sigma-Aldrich), was assessed by XRF in combination with high-resolution FESEM-EDS (probe current 1 nA), providing spectra and compositional data for impurity identification (Figure S6, Table S2). For electrical characterization, the recovered Ag powders were dispersed onto 3M tape to form conductive networks, and the bulk resistance was determined at Ag loadings of 2-6 mg using a two-point-probe digital multimeter (34465A, Keysight, USA).

2. STOICHIOMETRY AND LCA METHODOLOGY

To quantify reagent demand and potential gaseous nitrogen species generated under the EJSC, EJS and E-bath recycling conditions involving ten sequential cycles for the treatment of EoL SSCs, the accumulated dissolution masses of Ag and Al obtained from ICP-MS analysis (Section 1.5) were used as inputs to a stoichiometric framework. On this basis, theoretical upper limits of HNO_3 consumption and the evolution of nitric oxide (NO) and nitrous oxide (N_2O) were derived with reference to an initial electrolyte volume of 30 mL of 12 wt% HNO_3.

2.1. Reaction Stoichiometries of Metal Dissolutions in Dilute and Concentrated HNO_3

The calculations were established on the following representative dissolution stoichiometries of Ag and Al in dilute HNO_3:

Dissolution of Ag:

$$3\mathrm{Ag} + 4\mathrm{HNO_3} \rightarrow 3\mathrm{AgNO_3} + 2\mathrm{H_2O} + \mathrm{NO}\uparrow$$

Stoichiometric relation: 1 mol Ag corresponds to 4/3 mol HNO_3 and 1/3 mol NO.

Dissolution of Al:

$$8\mathrm{Al} + 30\mathrm{HNO_3} \rightarrow 8\mathrm{Al(NO_3)_3} + 15\mathrm{H_2O} + 3\mathrm{N_2O}\uparrow$$

Stoichiometric relation: 1 mol Al corresponds to 15/4 mol HNO_3 and 3/8 mol N_2O.

Note: Under dilute HNO_3, trace Cu^{2+} ions from the Ag paste have been reported to destabilize the protective Al_2O_3 layer and promote the formation of N_2O as the sole nitrogen oxide product.[12] Accordingly, Al dissolution is treated as producing N_2O in this work, while Ag dissolution yields NO under the same conditions.[13]

In addition to the electrochemical dissolution routes modelled using experimentally derived Ag and Al inputs under 12 wt% HNO_3, the 25 wt% HNO_3 reactions reported by Song et al.[6], who removed the Al backing prior to leaching, were included as a comparative reference to contextualize acid consumption and gaseous nitrogen emissions under conventional acid leaching conditions. This latter pathway represents a mechanically agitated system operating in moderately concentrated HNO_3, characteristic of established hydrometallurgical practice.

Dissolution of Ag (25 wt% HNO_3):

$$\mathrm{Ag} + 2\mathrm{HNO_3} \rightarrow \mathrm{AgNO_3} + \mathrm{H_2O} + \mathrm{NO_2}\uparrow$$

Stoichiometric relation: 1 mol Ag corresponds to 2 mol HNO_3 and 1 mol NO_2.

Note: Under moderately concentrated HNO_3, the oxidizing strength of the acid increases substantially due to the higher activity of nitrate and the reduced availability of protons for NO formation. Therefore, during conventional acid leaching, Ag dissolution generates NO_2 as the dominant gaseous nitrogen product.[6]

2.2. Stoichiometric Conversion to HNO_3 Consumption and Gaseous Nitrogen Emissions

The accumulated masses of Ag ($m_{\mathrm{Ag,accum}}$) and Al ($m_{\mathrm{Al,accum}}$), in mg, were converted to amounts of substance (n, in mmol) according to Eq. S7.

$$n_{\mathrm{Ag,accum}} = \frac{m_{\mathrm{Ag,accum}}}{M_{\mathrm{Ag}}};\ n_{\mathrm{Al,accum}} = \frac{m_{\mathrm{Al,accum}}}{M_{\mathrm{Al}}} \quad \text{(S7)}$$

where $M_{\mathrm{Ag}} = 107.87$ g/mol and $M_{\mathrm{Al}} = 26.98$ g/mol.

For reference, the initial 30 mL of 12 wt% HNO_3 contained 3.85 g of pure HNO_3.

First, the theoretical amount of HNO_3 consumed ($n_{\mathrm{HNO_3,cons}}$, in mmol), was then obtained from the dissolution stoichiometries, as expressed in Eq. S8.

$$n_{\mathrm{HNO_3,cons}} = \frac{4}{3} \cdot n_{\mathrm{Ag,accum}} + \frac{15}{4} \cdot n_{\mathrm{Al,accum}} \quad \text{(S8)}$$

The corresponding consumed mass of HNO_3 ($m_{\mathrm{HNO_3cons}}$) was then calculated via Eq. S9.

$$m_{\mathrm{HNO_3,cons}} = n_{\mathrm{HNO_3,cons}} \times M_{\mathrm{HNO_3}} \quad \text{(S9)}$$

where $M_{\mathrm{HNO_3}} = 63.01$ g/mol.

The equivalent volume of the 12 wt% HNO_3 stock solution ($V_{\mathrm{12\ wt\%\ HNO_3,cons}}$) was obtained using its mass fraction and density, as defined in Eq. S10.

$$V_{\mathrm{12\ wt\%\ HNO_3,cons}} = \frac{m_{\mathrm{HNO_3,cons}}}{0.12 \times \rho} \quad \text{(S10)}$$

where $\rho \approx 1.07$ g/mL.

Second, the theoretical molar yields of NO ($n_{\mathrm{NO,accum}}$) and N_2O ($n_{\mathrm{N_2O,accum}}$) were obtained from the dissolution stoichiometries, as given by Eqs. S11a–S11b.

$$n_{\mathrm{NO,accum}} = \frac{1}{3} \cdot n_{\mathrm{Ag,accum}} \quad \text{(S11a)}$$

$$n_{\mathrm{N_2O,accum}} = \frac{3}{8} \cdot n_{\mathrm{Al,accum}} \quad \text{(S11b)}$$

The corresponding masses of NO ($m_{\mathrm{NO,accum}}$) and N_2O ($m_{\mathrm{N_2O,accum}}$) were then calculated using their molar masses using Eqs. S12a–S12b.

$$m_{\mathrm{NO,accum}} = n_{\mathrm{NO,accum}} \times M_{\mathrm{NO}} \quad \text{(S12a)}$$

$$m_{\mathrm{N_2O,accum}} = n_{\mathrm{N_2O,accum}} \times M_{\mathrm{N_2O}} \quad \text{(S12b)}$$

where $M_{\mathrm{NO}} = 30.01$ g/mol and $M_{\mathrm{N_2O}} = 44.01$ g/mol.

Finally, the stoichiometry of the conventional acid leaching (25 wt% HNO_3) was incorporated, as shown in Eqs. S13a–S14b.

$$n_{\mathrm{HNO_3,cons}} = 2 \cdot n_{\mathrm{Ag,accum}} \quad \text{(S13a)}$$

$$n_{\mathrm{NO_2,accum}} = n_{\mathrm{Ag,accum}} \quad \text{(S13b)}$$

$$m_{\mathrm{HNO_3,cons}} = n_{\mathrm{HNO_3,cons}} \times M_{\mathrm{HNO_3}} \quad \text{(S14a)}$$

$$m_{\mathrm{NO_2,accum}} = n_{\mathrm{NO_2,accum}} \times M_{\mathrm{NO_2}} \quad \text{(S14b)}$$

where $M_{\mathrm{NO_2}} = 46.01$ g/mol. These relations were introduced to support the construction of the comparative process metrics and life-cycle assessment (LCA) indicators.

2.3. Uncertainty Propagation

The propagation of uncertainty for all derived quantities (Y), including $V_{\mathrm{HNO_3,cons}}$, $m_{\mathrm{NO,accum}}$ and $m_{\mathrm{N_2O,accum}}$, was rigorously accounted for using the law of propagation of uncertainty (LPU), the standard framework for error analysis in quantitative analytical measurements.[14] The standard deviations (SD, denoted as σ) associated with the ICP-MS-derived accumulated masses of Ag ($\sigma_{\mathrm{Ag,accum}}$) and Al ($\sigma_{\mathrm{Al,accum}}$) were treated as independent input variables.

For a calculated quantity Y that is a linear function of the measured masses:

$$Y = k_{\mathrm{Ag}} \cdot m_{\mathrm{Ag}} + k_{\mathrm{Al}} \cdot m_{\mathrm{Al}} \quad \text{(S15a)}$$

The absolute uncertainty was obtained using the LPU expression:

$$\sigma_Y = \sqrt{(k_{\mathrm{Ag}} \cdot \sigma_{\mathrm{Ag}})^2 + (k_{\mathrm{Al}} \cdot \sigma_{\mathrm{Al}})^2} \quad \text{(S15b)}$$

where k_{Ag} and k_{Al} are the stoichiometric conversion coefficients (e.g., per milligram of dissolved Ag or Al, the corresponding volumes of 12 wt% HNO_3 consumed and masses of NO or N_2O generated), respectively. The final values reported in the main text are expressed as mean ± 1 SD, with the SD representing the propagated measurement uncertainty σ_Y associated with the theoretically derived upper limits under the experimental conditions.

2.4. LCA Methodology

The LCA assessed the economic, resource, and environmental performance of the EJSC, EJS, and E-bath processes relative to conventional acid leaching. The analysis followed ISO 14040:2006 and ISO 14044:2006, using a unified functional unit and system boundary as defined in the main text. Inventory data were obtained from the ecoinvent 3.9.1 cutoff model, and impact assessment was performed using the ReCiPe 2016 Midpoint (H) method.

3. SUPPLEMENTARY FIGURES AND TABLES

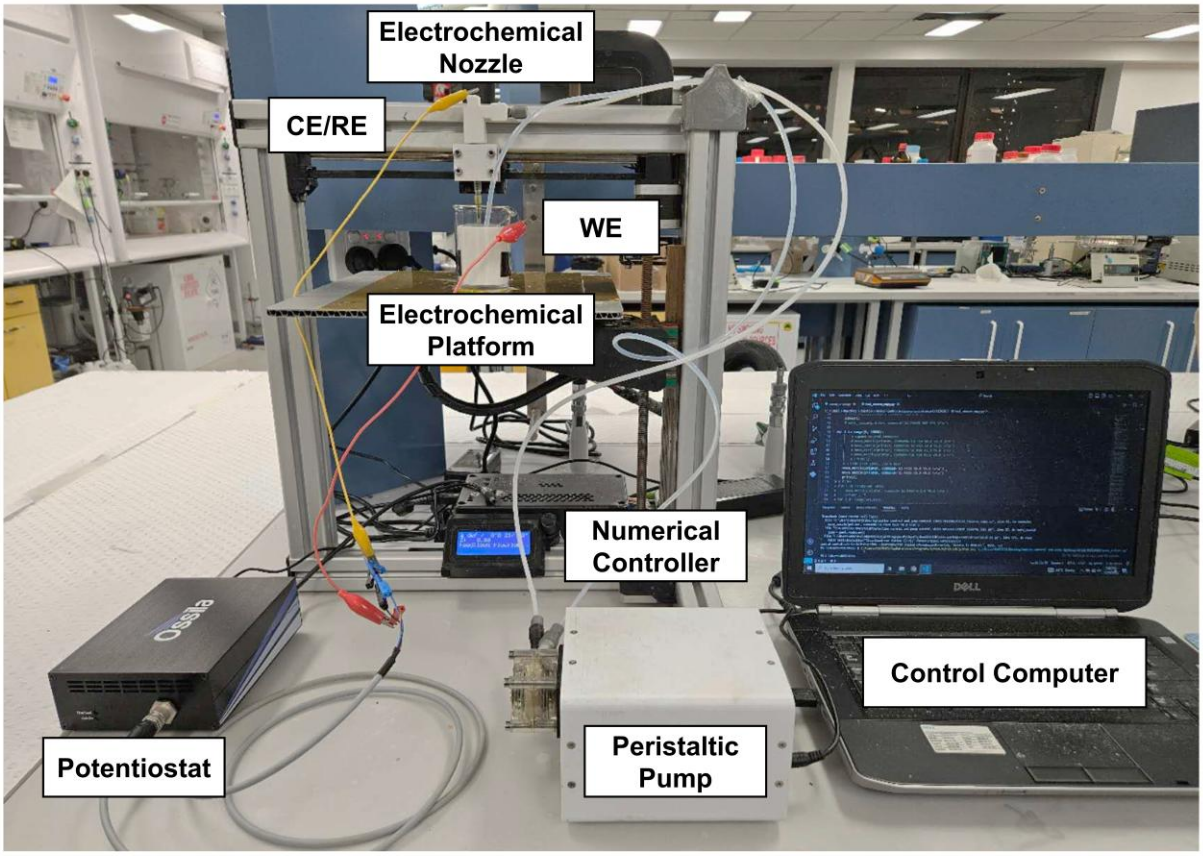

Figure S1. Photograph of the EJ-based recycling apparatus with labeled components, including the control computer (running G-code).

Table S1. ICP-MS quantification of Ag and co-dissolved metal impurities in leachates obtained from Ag reference acid leaching (70 wt% HNO_3, 48 h).

Ag reference acid leaching			
**Element**	**Mass (mg)**	**Element**	**Mass (mg)**
Ag	2.50 ± 0.04	Pb	0.04
Al	6.76	Cu	0.11
Ti	0.09	Mn	0.01
Bi	0.20	Zn	0.05
		**Metal impurities (mg)**	7.26

Note: Ag was measured in triplicate (±1 SD, n = 3), whereas impurity elements were determined once for reference.

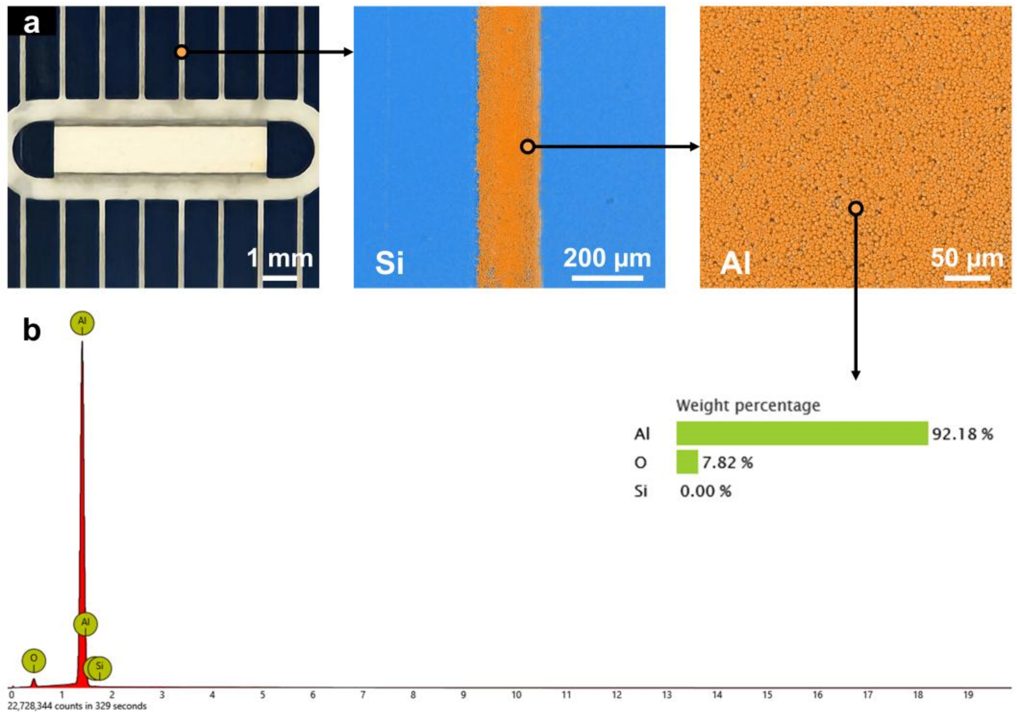

Figure S2. Confirmation of finger composition on pristine SSCs. (a) Optical and SEM-EDS images of the finger region. (b) EDS spectrum and compositional analysis showing a pure Al finger structure without detectable Ag.

Table S2. ICP-MS quantification of Ag mass balance for corresponding EJ recycling conditions in Figures 2–4.

**Recycling condition**	**Dissolved Ag (mg)**	**Residual Ag (mg)**	**Mass closure (%)**	**Data source**
EJS (2 V, 0.5 mm IEG, static)	0.95 ± 0.05	1.52 ± 0.03	99 ± 2.8	Fig. 2j
EJS (4 V, 0.5 mm IEG, static)	1.12 ± 0.07	1.34 ± 0.08	98 ± 3.3	Fig. 2j
EJS (6 V, 0.5 mm IEG, static)	1.34 ± 0.11	1.21 ± 0.06	102 ± 4.2	Fig. 2j
EJSC (2 V, 0.5 mm IEG, 6 mm/s)	1.46 ± 0.05	1.02 ± 0.04	99 ± 2.7	Fig. 2j, 3j
EJSC (2 V, 2.5 mm IEG, 6 mm/s)	1.59 ± 0.09	0.88 ± 0.02	99 ± 3.5	Fig. 3j
EJSC (2 V, 4.5 mm IEG, 6 mm/s)	1.73 ± 0.07	0.76 ± 0.05	100 ± 3.1	Fig. 3j, 4j
EJSC (2 V, 6.5 mm IEG, 6 mm/s)	1.24 ± 0.13	1.21 ± 0.07	98 ± 5.1	Fig. 3j
EJSC (2 V, 4.5 mm IEG, 7 mm/s)	1.93 ± 0.11	0.54 ± 0.06	99 ± 4.0	Fig. 4j
EJSC (2 V, 4.5 mm IEG, 8 mm/s)	1.70 ± 0.07	0.82 ± 0.02	101 ± 3.1	Fig. 4j
EJSC (2 V, 4.5 mm IEG, 9 mm/s)	1.44 ± 0.09	1.07 ± 0.08	100 ± 3.6	Fig. 4j

Notes:

(a) The initial Ag content was 2.50 ± 0.04 mg, determined by complete digestion of a pristine SSC in 70 wt% HNO_3 for 48 h (Table S1).

(b) Dissolved Ag refers to the Ag measured in the electrolyte collected after the EJ process.

(c) Residual Ag refers to the Ag remaining in the processed SSC substrate following digestion in 70 wt% HNO_3 for 24 h.

(d) Mass closure (%) = (Dissolved Ag + Residual Ag) / Initial Ag × 100%. Values are generally within 100 ± 5%, thereby confirming quantitative consistency.

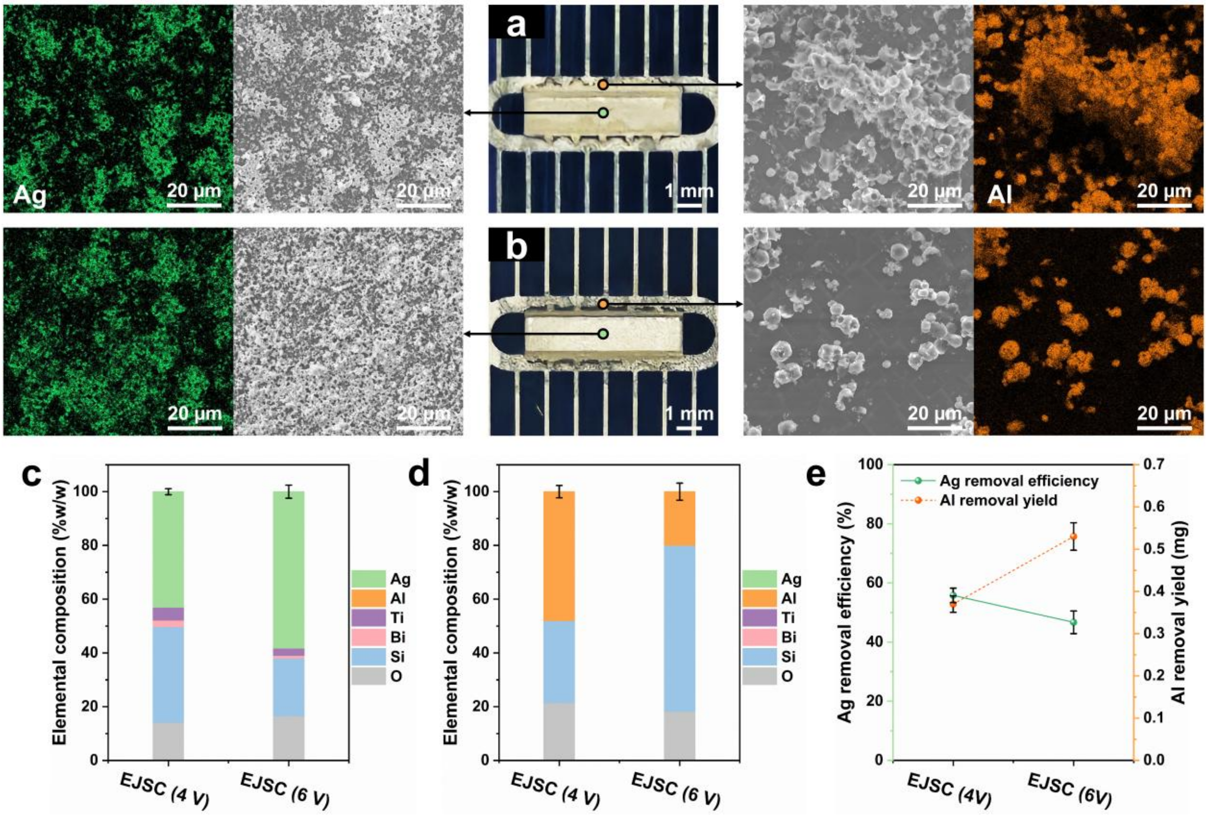

Figure S3. Effects of applied voltage on Ag extraction selectivity during EJSC (IEG: 0.5 mm; scanning velocity: 6 mm/s; reaction time: 2 min). (a, b) Optical and SEM-EDS images of the Ag pad center after EJSC at 4 and 6 V. (c, d) EDS analyses of (c) Ag pad center and (d) Al busbar, showing residual elemental distributions with increasing voltage. (e) ICP-MS quantification of Ag removal efficiency and Al dissolution yield. Error bars, where applicable, represent ±1 SD; n = 3.

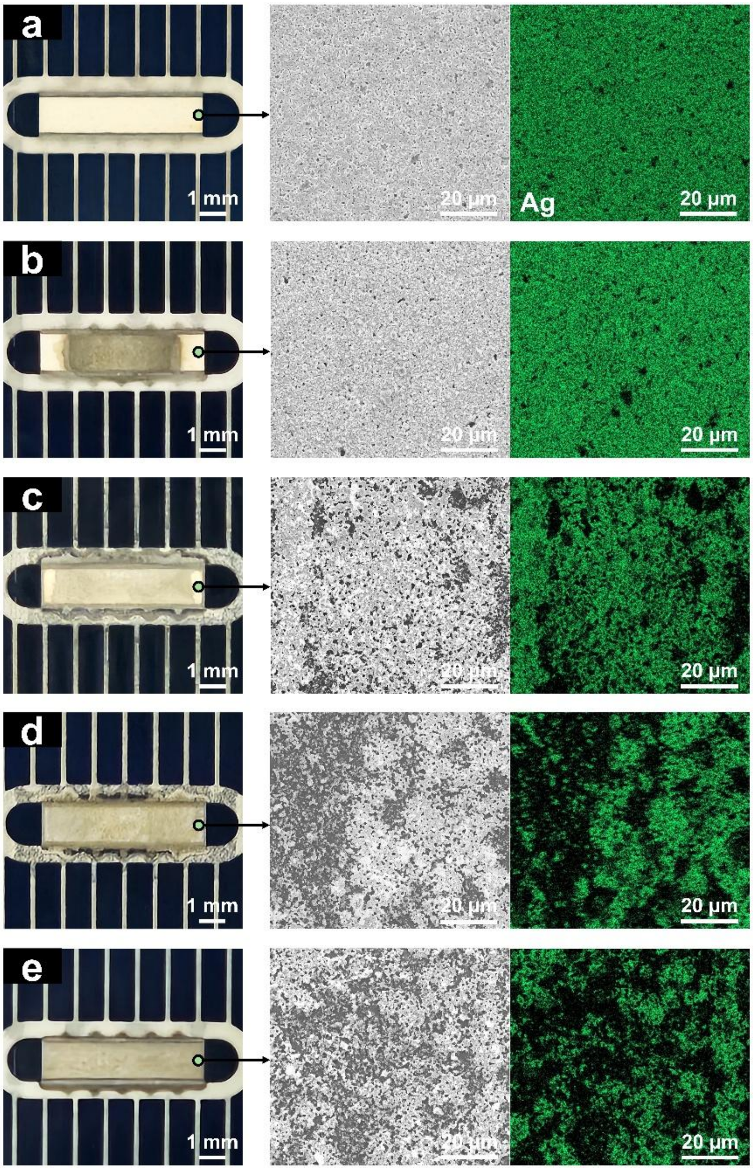

Figure S4. Effects of EJ-based recycling modes and applied voltages on the Ag pad edge (IEG: 0.5 mm; scanning velocity: 6 mm/s; reaction time: 2 min). (a-e) Optical and SEM-EDS images of SSCs: (a) pristine; (b-d) EJS at 2, 4, and 6 V; (e) EJSC at 2 V.

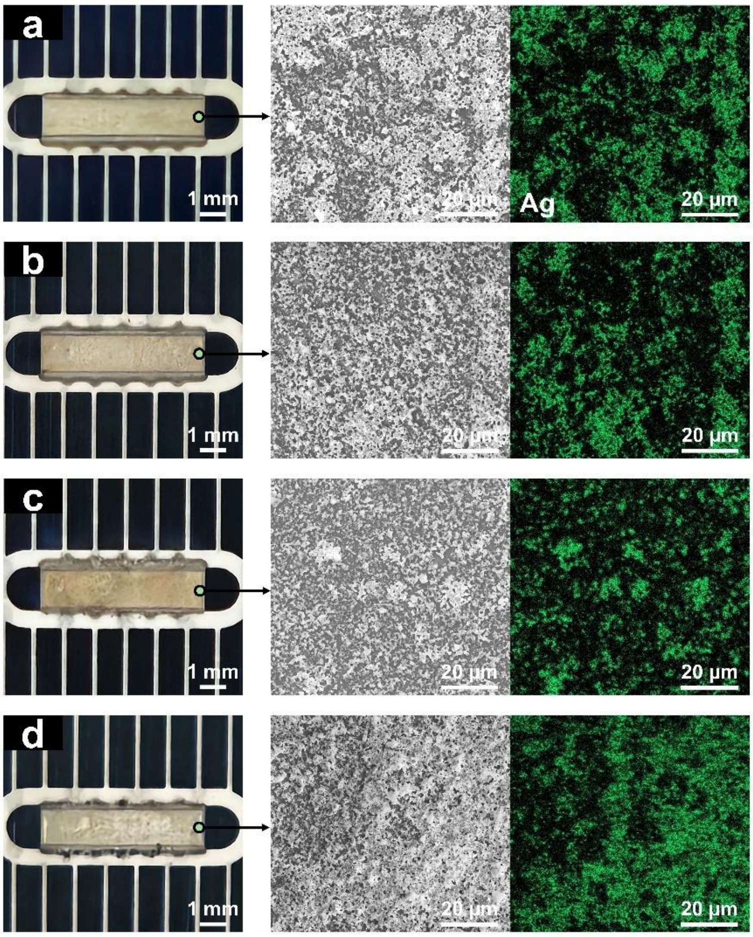

Figure S5. Effects of IEG on the Ag pad edge during EJSC (applied voltage: 2 V; scanning velocity: 6 mm/s; reaction time: 2 min). (a-d) Optical and SEM-EDS images of SSCs at IEGs of 0.5, 2.5, 4.5, and 6.5 mm.

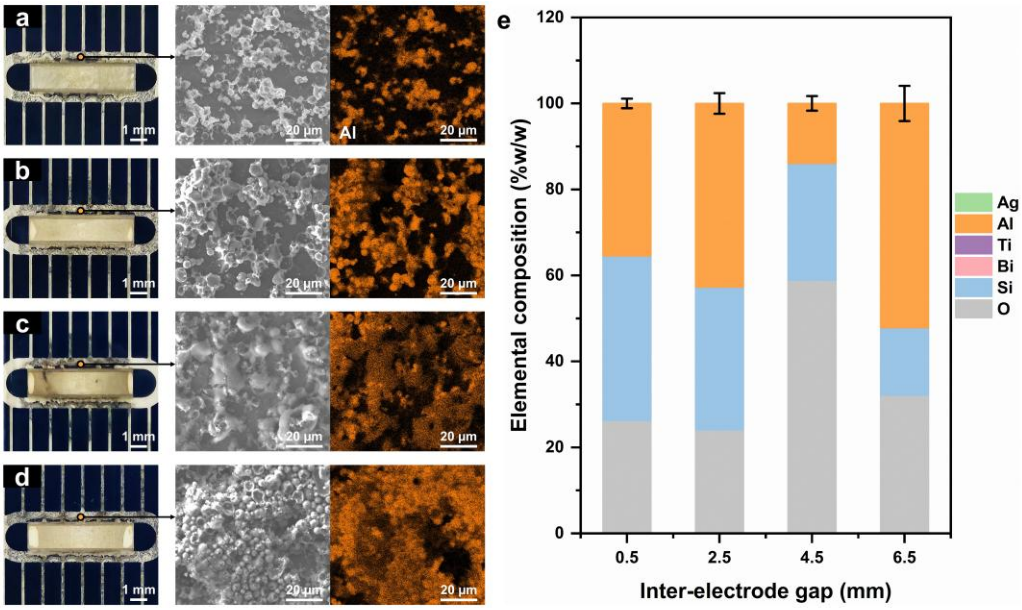

Figure S6. Effects of IEG on the Al busbar during EJS (applied voltage: 6 V; reaction time: 2 min). (a-d) Optical and SEM-EDS images of SSCs at IEGs of 0.5, 2.5, 4.5, and 6.5 mm. (e) EDS analyses of the Al busbar, showing residual elemental distributions as a function of IEG. Error bars, where applicable, represent ±1 SD; n = 3.

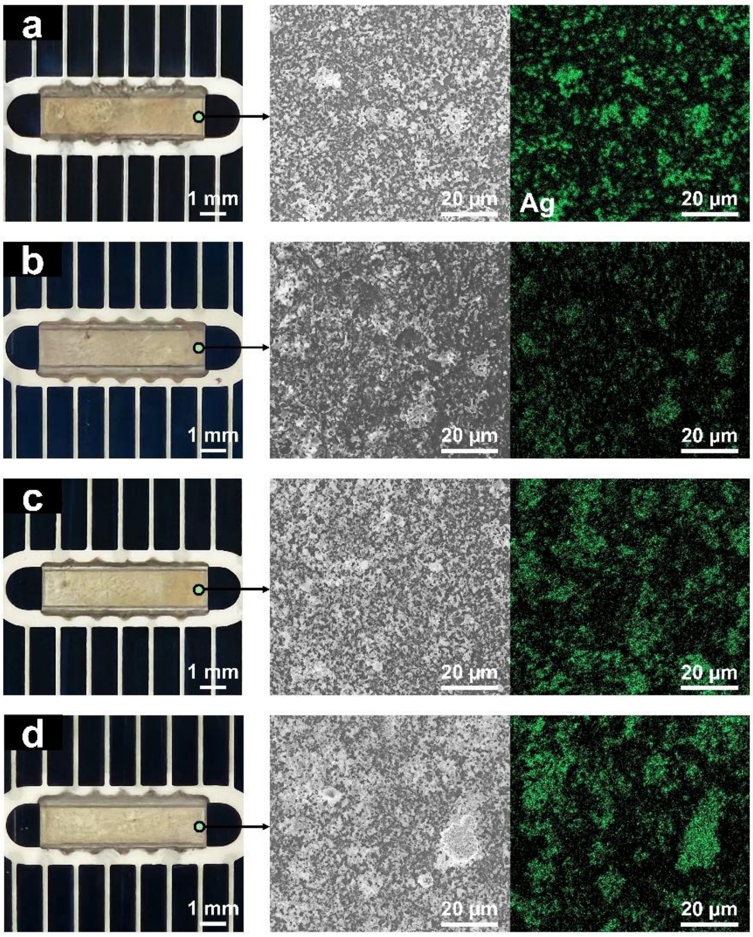

Figure S7. Effects of scanning velocity on the Ag pad edge during EJSC (applied voltage: 2 V; IEG: 4.5 mm; reaction time: 2 min). (a-d) Optical and SEM-EDS images of SSCs at scanning velocities of 6, 7, 8, and 9 mm/s.

Table S3. ICP-MS quantification of elemental metal contents in solutions obtained from EJSC, EJS, and E-bath recycling (4 min) and from benchmark acid leaching with 5 M HNO_3 solution (1 h, 200 rpm).

		**EJSC removal**	
**Element**	**Mass (mg)**	**Element**	**Mass (mg)**
Ag	2.43	Pb	-
Al	0.03	Cu	0.01
Ti	0.01	Mn	-
Bi	0.02	Zn	0.01
		**Metal impurities (mg)**	0.08
		**EJS removal**	
**Element**	**Mass (mg)**	**Element**	**Mass (mg)**
Ag	1.37	Pb	-
Al	0.06	Cu	-
Ti	-	Mn	-
Bi	0.01	Zn	-
		**Metal impurities (mg)**	0.07
		**E-bath removal**	
**Element**	**Mass (mg)**	**Element**	**Mass (mg)**
Ag	0.77	Pb	-
Al	0.05	Cu	-
Ti	-	Mn	-
Bi	-	Zn	-
		**Metal impurities (mg)**	0.05
		**Benchmark acid leaching**	
**Element**	**Mass (mg)**	**Element**	**Mass (mg)**
Ag	2.17	Pb	-
Al	0.68	Cu	0.01
Ti	0.06	Mn	-
Bi	0.01	Zn	0.01
		**Metal impurities (mg)**	0.77

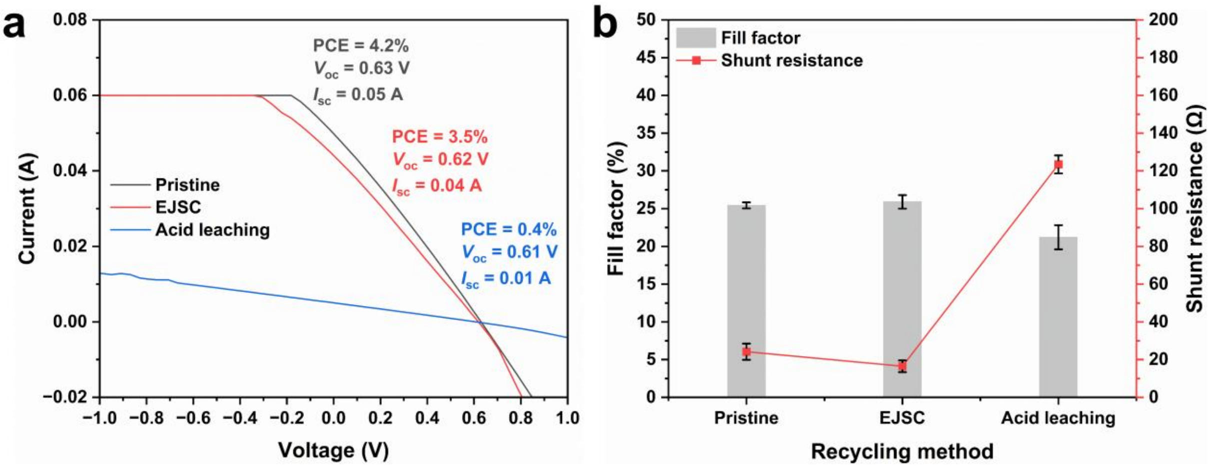

Figure S8. Comparative photovoltaic performance of EoL-SSCs before and after different recycling methods. (a) Current–voltage (I–V) characteristics of pristine, EJSC-treated, and acid-leached SSCs, illustrating that EJSC preserves the intrinsic photovoltaic response. (b) Fill factors and shunt resistances extracted from I–V measurements, showing that EJSC retains charge transport and passivation quality. Error bars, where applicable, represent ±1 SD; n = 3.

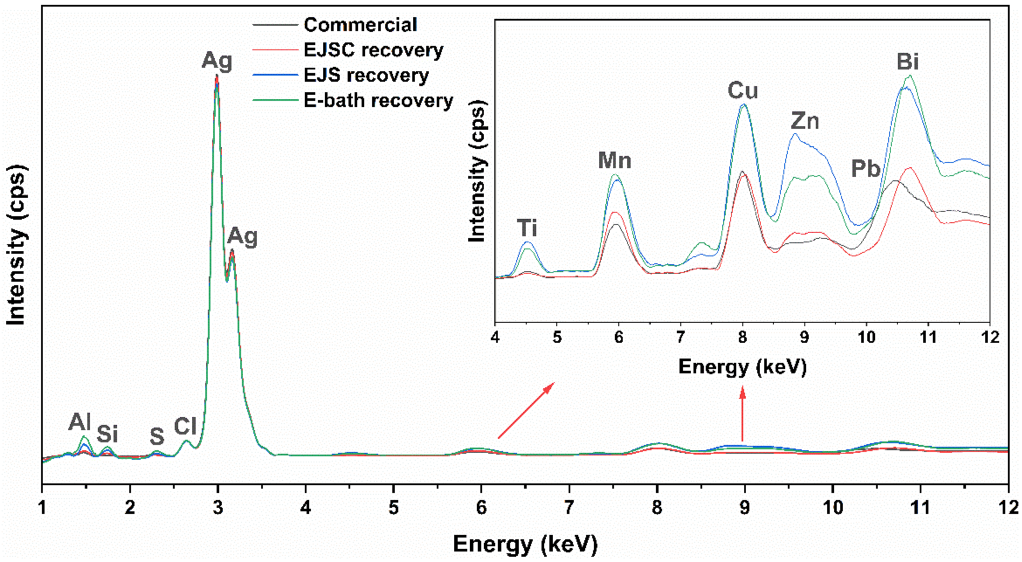

Figure S9. XRF spectra of Ag powders recovered by EJSC, EJS, and E-bath, benchmarked against commercial 3N-Ag. The spectra show a dominant Ag peak at 2.98 keV with only trace signals of Al, Si, Ti, Mn, Cu, Zn, Pb, and Bi. Weak S and Cl peaks are attributed to surface contaminants rather than intrinsic impurities.

Table S4. Purity profiles of commercial 3N-Ag and Ag powders recovered by EJSC, EJS, and E-bath (3 min), as determined by high-resolution FESEM-EDS.

**Commercial**			
**Element**	**Content (%w/w)**	**Element**	**Content (%w/w)**
Ag	99.91	Cu	0.03
Al	0.02	Zn	0.01
Ti	-	Pb	0.02
Si	-	Bi	-
Mn	0.01	**Total impurities (%w/w)**	0.09
**EJSC recovery**			
**Element**	**Content (%w/w)**	**Element**	**Content (%w/w)**
Ag	99.88	Cu	0.03
Al	0.03	Zn	0.01
Ti	-	Pb	-
Si	0.02	Bi	0.01
Mn	0.02	**Total impurities (%w/w)**	0.12
**EJS recovery**			
**Element**	**Content (%w/w)**	**Element**	**Content (%w/w)**
Ag	99.52	Cu	0.05
Al	0.15	Zn	0.07
Ti	0.05	Pb	-
Si	0.07	Bi	0.05
Mn	0.04	**Total impurities (%w/w)**	0.48
**E-bath recovery**			
**Element**	**Content (%w/w)**	**Element**	**Content (%w/w)**
Ag	99.46	Cu	0.05
Al	0.21	Zn	0.04
Ti	0.06	Pb	-
Si	0.08	Bi	0.05
Mn	0.05	**Total impurities (%w/w)**	0.54

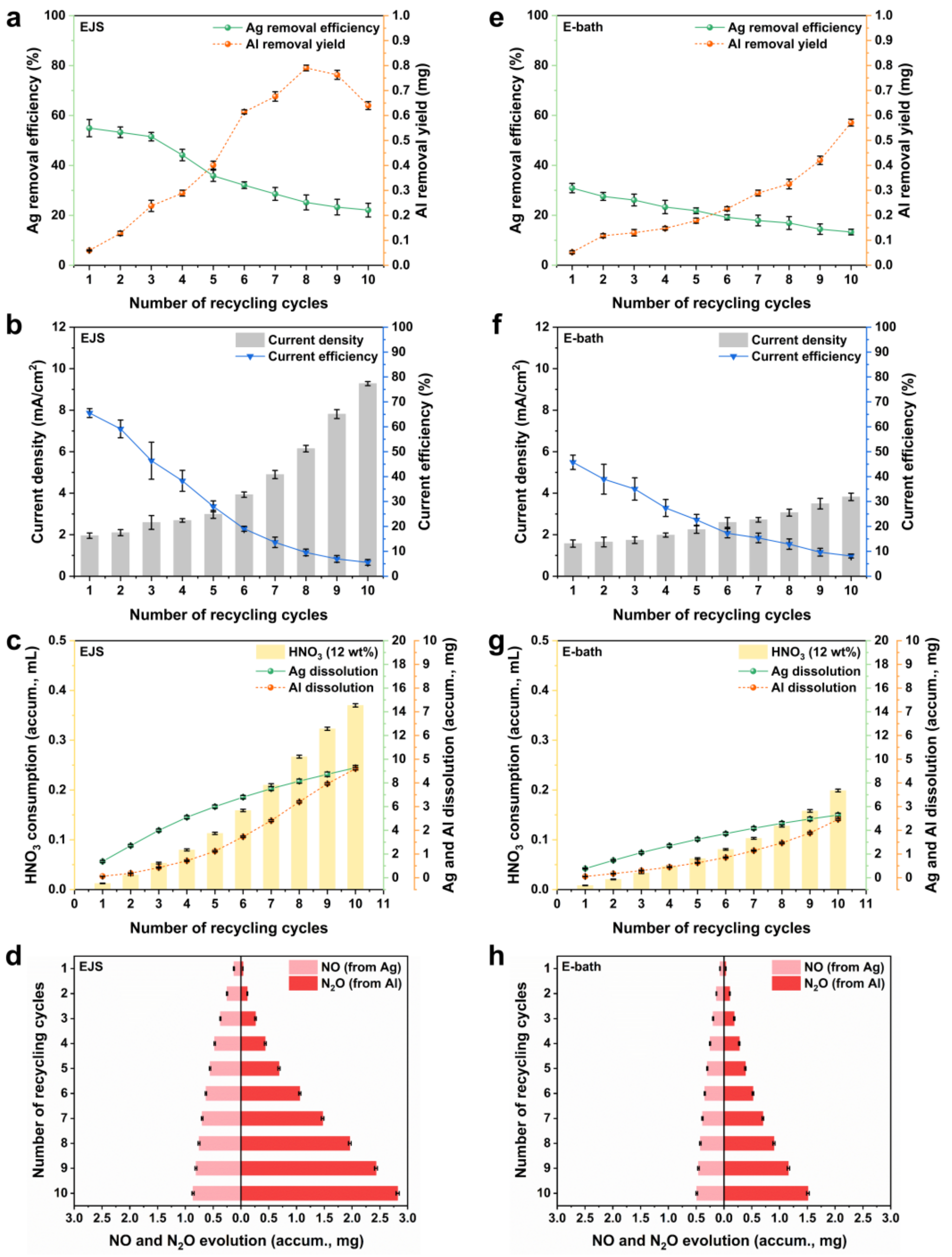

Figure S10. Multi-cycle performance of EJS and E-bath processes under identical electrochemical conditions (applied voltage: 2 V; IEG: 4.5 mm; reaction time per cycle: 4 min). (a-d) Cycle-resolved Ag/Al removal and electrochemical behavior, alongside cumulative acid consumption, metal dissolution, and gaseous byproduct release (NO and N_2O) during EJS recycling. (e-h) Corresponding results of the E-bath route for comparison. Error bars, where applicable, represent ±1 SD; n = 3.

Table S5. Life cycle inventory for the EJSC product system in Australia.

Flow	Category	Amount	Unit	Provider
**Inputs (Product - AU)**				
**Flow**	**Category**	**Amount**	**Unit**	**Provider**
EoL-SSC	Waste management	1.0	kg	-
Market HNO_3 (50 wt%)	Manufacturing	0.164	kg	Market for nitric acid - UN-Oceania
Recovered HNO_3 (50 wt%)	Manufacturing	6.566	kg	Wastewater treatment - AU
Electricity, medium voltage	Electricity supply	0.01162	kWh	Market for electricity - AU
**Outputs (Product - AU)**				
**Flow**	**Category**	**Amount**	**Unit**	**Provider**
Recovered Ag	Manufacturing	0.01241	kg	-
Photovoltaic cell, single-Si wafer	Manufacturing	0.7497	kg	-
Nitrate-containing wastewater	Waste management	6.7	kg	-
NO evolution	Emission to air	0.00115	kg	-
N_2O evolution	Emission to air	0.00026	kg	-
**Inputs (Wastewater treatment - AU)**				
**Flow**	**Category**	**Amount**	**Unit**	**Provider**
Nitrate-containing wastewater	Waste management	6.7	kg	-
Electricity, medium voltage	Electricity supply	5.77	kWh	Market for electricity - AU
**Outputs (Wastewater treatment - AU)**				
**Flow**	**Category**	**Amount**	**Unit**	**Provider**
Recovered HNO_3 (50 wt%)	Manufacturing	6.566	kg	-
Acidic residue	Emission to water	0.134	kg	-
NO_x capture	Emission to air	0.0	kg	-

Table S6. Life cycle inventory for the EJS product system in Australia.

Inputs (Product - AU)				
**Flow**	**Category**	**Amount**	**Unit**	**Provider**
EoL-SSC	Waste management	1.0	kg	-
Market HNO_3 (50 wt%)	Manufacturing	0.17	kg	Market for nitric acid - UN-Oceania
Recovered HNO_3 (50 wt%)	Manufacturing	6.560	kg	Wastewater treatment - AU
Electricity, medium voltage	Electricity supply	0.0076	kWh	Market for electricity - AU
**Outputs (Product - AU)**				
**Flow**	**Category**	**Amount**	**Unit**	**Provider**
Recovered Ag	Manufacturing	0.00594	kg	-
Nitrate-containing wastewater	Waste management	6.694	kg	-
NO evolution	Emission to air	0.00055	kg	-
N_2O evolution	Emission to air	0.00093	kg	-
**Inputs (Wastewater treatment - AU)**				
**Flow**	**Category**	**Amount**	**Unit**	**Provider**
Nitrate-containing wastewater	Waste management	6.694	kg	-
Electricity, medium voltage	Electricity supply	5.76	kWh	Market for electricity - AU
**Outputs (Wastewater treatment - AU)**				
**Flow**	**Category**	**Amount**	**Unit**	**Provider**
Recovered HNO_3 (50 wt%)	Manufacturing	6.56	kg	-
Acidic residue	Emission to water	0.13388	kg	-
NO_x capture	Emission to air	0.0	kg	-

Table S7. Life cycle inventory for the E-bath product system in Australia.

Flow	Category	Amount	Unit	Provider
**Inputs (Product - AU)**				
**Flow**	**Category**	**Amount**	**Unit**	**Provider**
EoL-SSC	Waste management	1.0	kg	-
Market HNO_3 (50 wt%)	Manufacturing	0.152	kg	Market for nitric acid - UN-Oceania
Recovered HNO_3 (50 wt%)	Manufacturing	6.578	kg	Wastewater treatment - AU
Electricity, medium voltage	Electricity supply	0.00549	kWh	Market for electricity - AU
**Outputs (Product - AU)**				
**Flow**	**Category**	**Amount**	**Unit**	**Provider**
Recovered Ag	Manufacturing	0.00324	kg	-
Nitrate-containing wastewater	Waste management	6.712	kg	-
NO evolution	Emission to air	0.0003	kg	-
N_2O evolution	Emission to air	0.00046	kg	-
**Inputs (Wastewater treatment - AU)**				
**Flow**	**Category**	**Amount**	**Unit**	**Provider**
Nitrate-containing wastewater	Waste management	6.712	kg	-
Electricity, medium voltage	Electricity supply	5.78	kWh	Market for electricity - AU
**Outputs (Wastewater treatment - AU)**				
**Flow**	**Category**	**Amount**	**Unit**	**Provider**
Recovered HNO_3 (50 wt%)	Manufacturing	6.578	kg	-
Acidic residue	Emission to water	0.13424	kg	-
NO_x capture	Emission to air	0.0	kg	-

Table S8. Life cycle inventory for the conventional acid leaching product system in Australia.

**Inputs (Product - AU)**				
**Flow**	**Category**	**Amount**	**Unit**	**Provider**
EoL-SSC	Waste management	1.0	kg	-
Market HNO_3 (50 wt%)	Manufacturing	0.2	kg	Market for nitric acid - UN-Oceania
Recovered HNO_3 (50 wt%)	Manufacturing	9.0	kg	Wastewater treatment - AU
Electricity, medium voltage	Electricity supply	1.168	kWh	Market for electricity - AU
**Outputs (Product - AU)**				
**Flow**	**Category**	**Amount**	**Unit**	**Provider**
Recovered Ag	Manufacturing	0.00862	kg	-
Solid residue	Waste management	0.99138	kg	-
Nitrate-containing wastewater	Waste management	9.18	kg	-
NO_2 evolution	Emission to air	0.00368	kg	-
**Inputs (Wastewater treatment - AU)**				
**Flow**	**Category**	**Amount**	**Unit**	**Provider**
Nitrate-containing wastewater	Waste management	9.18	kg	-
Electricity, medium voltage	Electricity supply	7.9	kWh	Market for electricity - AU
**Outputs (Wastewater treatment - AU)**				
**Flow**	**Category**	**Amount**	**Unit**	**Provider**
Recovered HNO_3 (50 wt%)	Manufacturing	9.0	kg	-
Acidic residue	Emission to water	0.18	kg	-
NO_x capture	Emission to air	0.0	kg	-
**Inputs (Solid residue treatment - AU)**				
**Flow**	**Category**	**Amount**	**Unit**	**Provider**
Solid residue	Waste management	0.99138	kg	-
Market cement (Portland)	Manufacturing	19.8276	kg	Market for cement - RoW
Tap water	Water supply	9.9138	kg	Market for tap water - RoW
Electricity, medium voltage	Electricity supply	0.093	kWh	Market for electricity - AU
**Outputs (Solid residue treatment - AU)**				
**Flow**	**Category**	**Amount**	**Unit**	**Provider**
Stabilized composite	Construction	30.7328	kg	-

Table S9. Comprehensive environmental impacts per kg of recovered Ag for different recycling methods.

**Impact category**	**Unit**	**EJSC**	**EJS**	**E-bath**	**Acid leaching**
Fossil depletion	kg oil-eq	116.04	242.42	444.44	487.24
Water consumption	m^3	1.53	3.23	5.71	8.09
Global warming	kg CO_2-eq	485.9	1015.15	1851.85	3271.46
Human carcinogenic toxicity	kg 1,4-DCB	41.74	87.04	159.88	128.77
Human non-carcinogenic toxicity	kg 1,4-DCB	805.8	1683.5	3086.42	2552.2
Fine particulate matter formation	kg $PM_{2.5}$-eq	0.52	1.07	1.94	2.78
Freshwater ecotoxicity	kg 1,4-DCB	21.92	45.79	83.95	73.32
Freshwater eutrophication	kg P-eq	0.73	1.52	2.79	1.87
Ionizing radiation	kBq Co-60-eq	0.85	1.79	3.24	15.55
Land use	m^2·a crop-eq	2.37	4.95	9.01	20.77
Marine ecotoxicity	kg 1,4-DCB	29.9	62.29	114.2	100.23
Marine eutrophication	kg N-eq	0.045	0.095	0.17	0.12
Mineral resource scarcity	kg Cu-eq	0.24	0.5	0.89	8.92
Ozone formation, human health	kg NO_x-eq	1.06	2.07	3.67	7.25
Ozone formation, terrestrial ecosystems	kg NO_x-eq	1.08	2.09	3.74	7.36
Stratospheric ozone depletion	kg CFC-11-eq	0.001	0.002	0.004	0.002
Terrestrial acidification	kg SO_2-eq	1.56	3.22	5.83	7.46
Terrestrial ecotoxicity	kg 1,4-DCB	356.97	747.48	1351.85	3399.07

REFERENCES

(1) Jung, B.; Park, J.; Seo, D.; Park, N. Sustainable System for Raw-Metal Recovery from Crystalline Silicon Solar Panels: From Noble-Metal Extraction to Lead Removal. *ACS Sustainable Chemistry & Engineering* **2016**, *4* (8), 4079-4083. DOI: 10.1021/acssuschemeng.6b00894.

(2) Dias, P.; Javimczik, S.; Benevit, M.; Veit, H.; Bernardes, A. M. Recycling WEEE: Extraction and concentration of silver from waste crystalline silicon photovoltaic modules. *Waste Management* **2016**, *57*, 220-225. DOI: https://doi.org/10.1016/j.wasman.2016.03.016.

(3) Chen, W.-S.; Chen, Y.-J.; Lee, C.-H.; Cheng, Y.-J.; Chen, Y.-A.; Liu, F.-W.; Wang, Y.-C.; Chueh, Y.-L. Recovery of Valuable Materials from the Waste Crystalline-Silicon Photovoltaic Cell and Ribbon. In *Processes*, 2021; Vol. 9, p 712.

(4) Luo, M.; Liu, F.; Zhou, Z.; Jiang, L.; Jia, M.; Lai, Y.; Li, J.; Zhang, Z. A comprehensive hydrometallurgical recycling approach for the environmental impact mitigation of EoL solar cells. *Journal of Environmental Chemical Engineering* **2021**, *9* (6), 106830. DOI: https://doi.org/10.1016/j.jece.2021.106830.

(5) Abdo, D. M.; El-Shazly, A. N.; Medici, F. Recovery of Valuable Materials from End-of-Life Photovoltaic Solar Panels. In *Materials*, 2023; Vol. 16, p 2840.

(6) Song, S.; Zhuo, Y.; Li, Q.; Shen, Y. Silver Recovery from Crystalline Silicon Photovoltaic Solar Cells Using Continuous Stirred-Tank Reactors. *Advanced Materials* **2024**, *36* (42), 2403653. DOI: https://doi.org/10.1002/adma.202403653.

(7) Gu, W.; Payne, D.; Veettil, B. P. Electrolyte-Jet 3D Printing of Copper-Based Strain Sensors for Physiological Signal Monitoring and Robotic Manipulation. *Advanced Materials Technologies n/a* (n/a), e00883. DOI: https://doi.org/10.1002/admt.202500883.

(8) Gu, W.; Marianov, A.; Jiang, Y. High-speed electrolyte jet 3D printing of ultrasmooth and robust Cu microelectrodes. *Journal of Materials Science* **2024**, *59* (11), 4605-4619. DOI: 10.1007/s10853-024-09514-7.

(9) Perera, R. T.; Rosenstein, J. K. Quasi-reference electrodes in confined electrochemical cells can result in in situ production of metallic nanoparticles. *Scientific Reports* **2018**, *8* (1), 1965. DOI: 10.1038/s41598-018-20412-2.

(10) Schuett, F. M.; Zeller, S. J.; Eckl, M. J.; Matzik, F. M.; Heubach, M.-K.; Geng, T.; Hermann, J. M.; Uhl, M.; Kibler, L. A.; Engstfeld, A. K.; et al. Versatile 3D-Printed Micro-Reference Electrodes for Aqueous and Non-Aqueous Solutions. *Angewandte Chemie International Edition* **2021**, *60* (42), 22783-22790. DOI: https://doi.org/10.1002/anie.202105871.

(11) Zheng, W.; Liu, M.; Lee, L. Y. S. Best Practices in Using Foam-Type Electrodes for Electrocatalytic Performance Benchmark. *ACS Energy Letters* **2020**, *5* (10), 3260-3264. DOI: 10.1021/acsenergylett.0c01958.

(12) Petruševski, V. M.; Bukleski, M.; Stojanovska, M. Reaction of aluminium with diluted nitric acid containing dissolved sodium chloride: on the nature of the gaseous products. *Chemistry* **2010**, *19* (3), 233-238.
(13) Sulcius, A. Reactions of Metals in Nitric Acid: Writing Equations and Calculating Electromotive Force of Redox Reaction. *Journal of Chemical Education* **2015**, *92* (12), 1971-1972. DOI: 10.1021/acs.jchemed.5b00328.
(14) Jcgm, J. Evaluation of measurement data—Guide to the expression of uncertainty in measurement. *Int. Organ. Stand. Geneva ISBN* **2008**, *50*, 134.
